# Combination of *Aspergillus niger* MJ1 with *Pseudomonas stutzeri* DSM4166 or mutant *Pseudomonas fluorescens* CHA0-*nif* improved crop quality, soil properties, and microbial communities in barrier soil

**DOI:** 10.3389/fmicb.2023.1064358

**Published:** 2023-02-02

**Authors:** Haiping Ni, Yuxia Wu, Rui Zong, Shiai Ren, Deng Pan, Lei Yu, Jianwei Li, Zhuling Qu, Qiyao Wang, Gengxing Zhao, Jianzhong Zhao, Lumin Liu, Tao Li, Youming Zhang, Qiang Tu

**Affiliations:** ^1^Helmholtz International Lab for Anti-Infectives, Shandong University–Helmholtz Institute of Biotechnology, State Key Laboratory of Microbial Technology, Shandong University, Qingdao, China; ^2^Qingdao Hexie Biotechnology Co., Ltd., Qingdao, China; ^3^CAS Key Laboratory of Quantitative Engineering Biology, Shenzhen Institute of Synthetic Biology, Shenzhen Institute of Advanced Technology, Chinese Academy of Sciences, Shenzhen, China; ^4^Shandong Agricultural Technology Extension Center, Jinan, China; ^5^National Engineering Laboratory for Efficient Utilization of Soil and Fertilizer, College of Resources and Environment, Shandong Agricultural University, Tai’an, China; ^6^Shandong Rural Economic Management and Service Center, Jinan, China

**Keywords:** phosphate-solubilizing, biological nitrogen fixation, fertilizer reduction, bacterial community, green

## Abstract

Soil salinization and acidification seriously damage soil health and restricts the sustainable development of planting. Excessive application of chemical fertilizer and other reasons will lead to soil acidification and salinization. This study focus on acid and salinized soil, investigated the effect of phosphate-solubilizing bacteria, *Aspergillus niger* MJ1 combined with nitrogen-fixing bacteria *Pseudomonas stutzeri* DSM4166 or mutant *Pseudomonas fluorescens* CHA0-*nif* on crop quality, soil physicochemical properties, and microbial communities. A total of 5 treatments were set: regular fertilization (T1), regular fertilization with MJ1 and DSM4166 (T2), regular fertilization with MJ1 and CHA0-*nif* (T3), 30%-reducing fertilization with MJ1 and DSM4166 (T4), and 30%-reducing fertilization with MJ1 and CHA0-*nif* (T5). It was found that the soil properties (OM, HN, TN, AP, AK, and SS) and crop quality of cucumber (yield production, protein, and vitamin C) and lettuce (yield production, vitamin C, nitrate, soluble protein, and crude fiber) showed a significant response to the inoculated strains. The combination of MJ1 with DSM4166 or CHA0-*nif* influenced the diversity and richness of bacterial community in the lettuce-grown soil. The organismal system-, cellular process-, and metabolism-correlated bacteria and saprophytic fungi were enriched, which were speculated to mediate the response to inoculated strains. pH, OM, HN, and TN were identified to be the major factors correlated with the soil microbial community. The inoculation of MJ1 with DSM4166 and CHA0-*nif* could meet the requirement of lettuce and cucumber growth after reducing fertilization in acid and salinized soil, which provides a novel candidate for the eco-friendly technique to meet the carbon-neutral topic.

## Introduction

Soil salinization and acidification are commonly seen as soil barrier that breaks soil health and restricts the sustainable development of planting. According to the statistical data, the area of global saline-alkali land has reached 9.55 × 10^8^ hm^2^, and the area in China is about 9.91 × 10^7^ hm^2^. The micropores of the saline-alkali soil are large containing a variety of soluble salts, including chloride, sulfate, carbonate, calcium, magnesium, potassium ions, etc. Meanwhile, insufficient drainage would result in the accumulation of salt ions. High concentrations of salt ions could affect the soil structure, reduce the penetration of water into the soil pores, and therefore induced soil compaction. In addition, high concentrations of salt ions in the soil will reduce the relative activity of mineral elements, thereby reducing their uptake by plants and affecting plant growth. On the other hand, soil acidification is also a challenging problem for global agriculture. The long-term and excessive application of chemical fertilizers, especially the excessive application of nitrogen, has become one of the important reasons for the aggravation of soil acidification. Studies have found that long-term NPK fertilizers can improve soil comprehensive fertility and economic yield, but at the same time led to soil acidification (Zhu et al., 2018; Bai et al., 2020; Yan et al., 2020; Aasfar et al., 2021). Soil acidification results in a series of hazards. First, the uneven distribution of soil nutrients leads to poor growth of above-ground crops during planting, which reduces the productivity and economic benefits. Meanwhile, great damages were exerted in soil micro-ecosystems and other functions. There was a lack of scientific guidance for the application of organic fertilizers, which is also another fatal factor resulting in the large difference in the fertilizer effects. Exploring novel fertilizer strategies to improve the productivity of barrier soil and the production of crops is of urgent need.

Phosphorus in the soil is easily integrated with metal ions, such as calcium, iron, and aluminum, and formed insoluble metal minerals, which results in the reduced availability of phosphorus and further limits its uptake by plants (Wu et al., 2019). The level of total phosphorus in the soil is always found to be much higher than available phosphorus, and in order to improve the yield, a huge number of phosphate fertilizer was applied to the soil. According to previous data, less than 20% phosphate fertilizer can be used by crops, therefore leading to the increasing accumulation of phosphorus in the soil (Li et al., 2015). Excessive phosphorus in the soil could enter the aquatic system through runoff, inducing eutrophication and aggravating non-point pollution. Moreover, excessive phosphorus is also a major inducing factor of soil acidification, due to the fact that some kinds of phosphate fertilizer like calcium superphosphate, could produce free acid into the soil, which results in the decreasing soil pH. Therefore, the conversion of insoluble phosphorus into soluble forms and reducing excessive accumulation of phosphorus in soil are of urgent need to be solved.

Phosphate-solubilizing microorganisms (PSMs) in the soil could transform the insoluble P into the form that can be utilized by plants. Hitherto, numerous PSMs have been isolated from different kinds of soil, and the number of fungi was larger than that of bacteria. The mechanism of phosphate solubilization by PSMs varies from different strains, including the secretion of phytases, nucleases, phosphatases, and organic acid (Rawat et al., 2021). *Aspergillus niger* MJ1 is a PSM isolated from alkaline soil, which was demonstrated to improve the content of available phosphorus through dissolving organic and inorganic phosphorus (Schneider et al., 2010; Xiao et al., 2015; Klaic et al., 2021). Therefore, the application of *A. niger* MJ1 might ameliorate the deficiency of phosphorus in the soil and benefit crop production.

With the increasing food requirement, the amount of nitrogen fertilizers applied in agriculture also grew rapidly. However, the utilization of nitrogen fertilizers is unsatisfactory. According to recent data, only 30–50% of the applied fertilizers can be utilized, and the residuals were left in the soil (Xu et al., 2012; Wang et al., 2018). Excessive nitrogen would induce a series of soil degradation problems, such as soil acidification and compaction. Biological nitrogen fixation is one of the major sources of soil nitrogen, where N_2_ was reduced to ammonia by nitrogen-fixing microorganisms. The nitrogen-fixing (*nif*) gene cluster is a typical nitrogen fixing-correlated gene that encodes the components of the nitrogenase enzyme and catalyzes the biological nitrogen fixation process (Curatti and Rubio, 2014; Thiel, 2019). Previous studies have identified a series of diazotrophs with various genetic components and arrangements of *nif* genes, such as *Paenibacillus brasilensis* PB24, *Klebsiella oxytoca* M5a1, and *Azotobacter vinelandii* AvOP (Mahmud et al., 2020; do Carmo Dias et al., 2021). Increasing studies have been devoted to investigating the genetics and regulation of *nif* genes in some functional bacteria that play roles in the circulation of other elements. For instance, in *Leptospirillum ferrooxidans*, an acidophile iron-oxidizing bacteria, a variety of nitrogen fixation-correlated genes were identified, including the specific member of the *nif* family, *nifA*, indicating its potential in biological nitrogen fixation (Parro and Moreno-Paz, 2004). *Pseudomonas stutzeri* DSM4166 was identified as a diazotroph isolated from the rhizosphere of a Sorghum nutans cultivar (Yu et al., 2011). In our previous work, we have established a mutant strain of *Pseudomonas fluorescens* CHA0 through integrating a *nif* cluster with a length of 49 kb from *P. stutzeri* DSM4166 into the genome of *P. fluorescens* CHA0 by biparental conjugation, which makes *P. fluorescens* CHA0 possess the potential ability of nitrogen fixation (Yu et al., 2019; Jing et al., 2020).

Soil microorganisms play vital roles in regulating the physiochemical properties and biophysical processes of the soil, which would indirectly influence plant growth. The structure of the soil microbial community could disclose the mechanism of fertilization application and indicate the healthy condition of the soil. In previous studies, the soil bacterial community was suggested to be sensitive to the soil nutrients as well as some exogen pollutants and fertilization, such as biochar, chemical fertilizers, and organic fertilizers (Balkhair, 2016; Zhang et al., 2019, 2021). Hence, the addition of PSMs or diazotrophs might affect the composition and structure of soil bacteria, which could provide more functional bacteria and reveal the mechanism underlying the applied bacteria.

This study aimed to clarify the following questions: (1) evaluating the effect of the combination of *Aspergillus niger* MJ1, *P. stutzeri* DSM4166, and *P. fluorescens* CHA0-nif mutant strain on the quality of lettuce and cucumber and the properties of barrier soil; (2) revealing the response of soil bacteria to the application of modified fertilization strategies; (3) exploring the optimal strain combination to provide the theoretical basis for improving production and quality of lettuce and cucumber.

## Materials and methods

### Field experiment

The field experiment was conducted in Haofeng base, Jimo District, Qingdao, Shandong Province, China (36.38°N, 120.33°E). The acid field was grown with lettuce (pH 5.5–6.1) and the salinization field was grown with cucumber (pH 7.6–7.9, soluble salt 0.14–0.19%). The lettuce (*Lactuca sativa* L. planting density 3,300 trees per mu) was transplanted at the four-leave stage on 21st, August 2020, while the cucumber (*Cucumis sativus* L. planting density 3,500 trees per mu) was seeded on 22nd, June 2020. The quality and production of the plants were added up on the 9th, October 2020, and 20th, August 2020, respectively. The soil samples were also collected at different layers (0–20 and 20–40 cm) at the same time.

The completely randomized block design was applied with five treatments and three triplicates of each. Each block possessed an area of 30 m^2^ (length of 6 m and width of 5 m). The specific application fertilization and strains of each treatment were summarized in Table 1. Regular fertilization was composed of 675 kg/ha. The microbial inoculants were used with a concentration of 600 L/ha. No fertilization in other periods.

**Table 1:** The fertilization and strain application of each treatment.

		Regular fertilization	*Aspergillus niger* MJ1	*Pseudomonas stutzeri* DSM4166	*Pseudomonas fluorescens* CHA0-*nif*
Lettuce	L1	+	−	−	−
	L2	+	+	+	−
	L3	+	+	−	+
	L4	Reduce 30%	+	+	−
	L5	Reduce 30%	+	−	+
Cucumber	C1	+	−	−	−
	C2	+	+	+	−
	C3	+	+	−	+
	C4	Reduce 30%	+	+	−
	C5	Reduce 30%	+	−	+

Earthworm surveys were conducted using cubes of soil (length 1.0 m, width 2.0 m, and height 0.3 m) excavated manually in the field after harvest, all earthworms were counted.

### Strains

The *A. niger* MJ1 was isolated from the from alkaline soil, which had been deposited in CCTCC (China Center for Type Culture Collection) on 5th, January 2015 (CCTCC No: M2015004), routinely solid cultivated with aeration at 30°C in the PDA medium (pH 7.0, Potato 200 g/L, Dextrose 20 g/L, and Agar 20 g/L). The wild-type strain *P. stutzeri* DSM4166 (deposited in DSMZ: Deutsche Sammlung von Mikroorganismen und Zellkulturen, No: DSM4166) and its derived strain CHA0-*nif* (deposited in CGMCC: China General Microbiological Culture Collection Center on 31st, July 2017, No: CGMCC 14476) used in this study were routinely cultivated with aeration at 30°C in the KB medium (pH 7.0, K_2_HPO_4_ 1.5 g/L, MgSO_4_·7H_2_O 1.5 g/L, peptone 20 g/L, glycerin 10 ml/L). At least 200 million colony-forming units per ml (CFU/ml) of *P. stutzeri* DSM4166 or CHA0-*nif* were added to the soil during the sowing of the crop’s seeds. While 5 million CFU/ml of *A. niger* MJ1 spore powder was applied to the soil.

### Soil and plant analysis

The major physicochemical properties of collected soil samples, including pH, organic matter (OM), hydrolysis nitrogen (HN), total nitrogen (TN), available phosphorus (AP), available potassium (AK), soluble salt (SS), microbial biomass carbon (MBC), microbial biomass nitrogen (MBN), and microbial biomass phosphorus (MBP) were analyzed with regular methods according to previous reports.

The total weight of the cucumber and lettuce was evaluated after the harvest and converted to the yield per hectare. The weight and height of lettuce heads and the characteristics of the lettuce vanes (length and width) were measured to estimate the lettuce quality. The soluble protein, sugar, and nitrates in lettuce were analyzed according to reported methods (Aasfar et al., 2021). The content of vitamin C was assessed according to Li et al. with a UV-spectrophotometer (Sánchez-Rangel et al., 2013).

### DNA isolation and sequencing

DNA extraction was carried out with the E.Z.N.A. soil kit (Omega Bio-Tek, United States) according to the manufacturer’s protocols. After evaluating the purity and concentration of isolated DNA with NanoDrop 2000 (Thermo Fisher, United States), the amplification process was conducted by PCR system. The primer sequences are as follows: 338F 5′-ACTCCTACGGGAGGCAGCAG-3′ and 806r 5′-GGACTACHVGGGTWTCTAAT-3′ for bacterial V3–V4 region of bacterial 16S rRNA gene, ITS1F 5′-CTTGGTCATTTAGAGGAAGTAA-3′ and ITS2R 5′-GCTGCGTTCTTCATCGATGC-3′ for fungal ITS hypervariable regions. The Illumina Miseq sequencing process was performed on an Illumina Miseq platform (Illumina, United States) by Novogene Bioinformatics Technology Co. (Beijing, China) according to the standard protocols. The quality control of raw data was conducted with Trimmomatic and merged by FLASH.

The Illumina sequencing was performed with a MiSeq PE250 sequencer (Illumina, United States). The USEARCH method (Edgar et al., 2011) was applied for the stitching, filtering, deduplication, and clustering of the data. With the help of the UPARSE algorithm, the sequences with similarity of over 97% were classified into the same OTU. Then, the sequences were classified and annotated to establish the taxonomic tree and phylogenetic tree. The alpha- and beta-diversity indexes were calculated with the rarefied OTUs. The alpha-diversity index includes Chao1, species, Shannon, Simpson, Faith, Pielou, and coverage, which were calculated by the R-package in QIIME2. For the further beta-diversity analysis, the principal coordinate analysis (PCoA) was conducted with four distance algorithms.

### Statistical analysis

The obtained data were represented as mean value ± SD and analyzed by SPSS 26.0 software. The difference between groups was evaluated with one-way ANOVA followed by the Duncan *post-hoc* test. The microbial data were analyzed with the R software (Version 2.15.3) including the following parts. The alpha-diversity was estimated by the Ace and Shannon index and compared with the ANOVA. The beta-diversity was assessed through the principal coordinate analyses based on weighted and unweighted unifrac distance algorithms. The Lefse analysis was performed to identify differential microbial communities or species between different treatments. The correlation between soil physicochemical properties and microbial community was analyzed with RDA analysis. The function analysis was also performed to further predicted the potential functional role of identified microorganisms. PICRUSt is a bioinformatics tool that uses marker genes to predict the functional content of microorganism. In this study, this method was employed to predict the potential functions of each sample based on 16S rRNA sequencing data. We used the KEGG database and performed closed reference OTU picking using the sampled reads against a Greengenes reference taxonomy (Greengenes database Version). The 16S copy number was then normalized, molecular functions were predicted and final data were summarized into KEGG pathways. *p* < 0.05 indicates statistical significance.

## Results

### Effect of inoculated strains on barrier soil properties

Compared with regular fertilization, the inoculation of the studied strains dramatically influenced the OM, HN, TN, AP, AK, and SS contents in the barrier soil with various depths. The detailed soil physiochemical properties are summarized in Table 2.

**Table 2:** Basic physicochemical properties of soil at different depths grown with cucumber or lettuce.

Crop	Depth	Group	pH	OM (g/kg)	HN (mg/kg)	TN (g/kg)	AP (mg/kg)	AK (mg/kg)	SS (%)
Cucumber	0–20 cm	C1	7.75 ± 0.10a	20.28 ± 0.54a	150.07 ± 4.11a	1.07 ± 0.10c	49.08 ± 0.79b	278.70 ± 11.06c	0.183 ± 0.012a
		C2	7.71 ± 0.06a	20.43 ± 2.03a	155.07 ± 9.30a	1.53 ± 0.04b	61.50 ± 7.30a	417.10 ± 12.10a	0.173 ± 0.017a
		C3	7.71 ± 0.08a	20.97 ± 1.23a	159.86 ± 6.04a	1.74 ± 0.19a	63.24 ± 5.51a	439.33 ± 27.49a	0.168 ± 0.005a
		C4	7.67 ± 0.10a	20.47 ± 0.75a	150.36 ± 3.10a	1.41 ± 0.05b	49.55 ± 0.50b	341.80 ± 15.33b	0.176 ± 0.019a
		C5	7.70 ± 0.12a	20.32 ± 0.22a	150.76 ± 5.69a	1.42 ± 0.12b	50.56 ± 1.77b	348.70 ± 34.25b	0.173 ± 0.004a
	20–40 cm	C1	7.86 ± 0.09a	12.12 ± 0.24a	103.27 ± 3.74c	0.94 ± 0.04c	28.50 ± 6.78b	236.20 ± 16.20c	0.167 ± 0.018a
		C2	7.82 ± 0.10a	12.85 ± 1.63a	119.04 ± 1.58b	1.19 ± 0.10ab	39.70 ± 4.41a	335.10 ± 11.62b	0.142 ± 0.010a
		C3	7.76 ± 0.10a	13.20 ± 0.76a	128.24 ± 5.05a	1.27 ± 0.05a	37.53 ± 2.09a	387.00 ± 2.20a	0.141 ± 0.014a
		C4	7.85 ± 0.14a	12.56 ± 0.52a	114.24 ± 5.28b	1.12 ± 0.11b	28.24 ± 4.43b	243.37 ± 31.00c	0.147 ± 0.018a
		C5	7.84 ± 0.05a	12.80 ± 0.25a	104.16 ± 4.62c	1.10 ± 0.06b	28.37 ± 5.01b	256.25 ± 14.45c	0.145 ± 0.020a
Lettuce	0–20 cm	L1	5.53 ± 0.11a	13.71 ± 0.47b	109.92 ± 6.30b	0.98 ± 0.02b	67.84 ± 3.65c	208.97 ± 10.55b	0.061 ± 0.006a
		L2	5.74 ± 0.29a	13.66 ± 0.37b	123.81 ± 6.48a	1.01 ± 0.05b	91.29 ± 2.43a	184.80 ± 13.55b	0.057 ± 0.001ab
		L3	5.60 ± 0.17a	14.84 ± 0.57a	121.24 ± 3.98a	1.12 ± 0.12a	78.00 ± 4.48b	387.00 ± 2.20a	0.057 ± 0.004ab
		L4	5.87 ± 0.24a	13.78 ± 0.61b	109.57 ± 6.38b	0.96 ± 0.03b	75.72 ± 5.28bc	205.62 ± 25.32b	0.053 ± 0.001b
		L5	5.78 ± 0.10a	13.94 ± 0.33b	105.49 ± 3.45b	0.95 ± 0.03b	77.30 ± 5.64b	192.77 ± 13.54b	0.054 ± 0.004ab
	20–40 cm	L1	5.95 ± 0.09a	11.74 ± 0.60a	86.94 ± 4.30d	0.87 ± 0.02b	67.97 ± 7.50b	166.00 ± 2.57b	0.050 ± 0.003a
		L2	6.00 ± 0.12a	12.15 ± 0.89a	94.99 ± 4.04bc	1.00 ± 0.17b	83.09 ± 5.31a	164.17 ± 11.91b	0.049 ± 0.006a
		L3	6.08 ± 0.05a	13.20 ± 0.76a	128.24 ± 5.05a	1.27 ± 0.05a	67.53 ± 2.09b	195.03 ± 9.11a	0.048 ± 0.002a
		L4	6.03 ± 0.14a	11.82 ± 0.76a	95.81 ± 3.16b	0.87 ± 0.02b	67.25 ± 3.32b	193.05 ± 0.75a	0.045 ± 0.005a
		L5	6.06 ± 0.09a	11.82 ± 1.09a	87.76 ± 4.57cd	0.87 ± 0.03b	75.42 ± 6.57ab	160.53 ± 14.35b	0.045 ± 0.007a

Different letters indicate significant difference.

Specifically, in the 0–20 cm of the salinized soil with cucumber, the MJ1 significantly improved the content of TN, AP, and AK. Meanwhile, the co-inoculation of CHA0-*nif* showed a significant promoted effect on TN in comparison with the co-inoculation of DSM4166. After reducing 30% of the regular fertilization, the inoculation of MJ1 with DSM4166 or CHA0-*nif* increased the soil content of TN, AP, and AK relative to treatment with regular fertilization, and no significant difference was observed between the DSM4166 and CHA0-*nif*. For the 20–40 cm soil, the contents of HN, TN, AP, and AK were found to elevate in the presence of MJ1 co-inoculated with DSM4166 and CHA0-*nif*, and the enhanced effect of CHA0-*nif* was significantly stronger than DSM4166 in the content of HN and AK. The TN in the soil with co-inoculation of MJ1 and DSM4166 or CHA0-*nif* was significantly higher than the regular fertilization, and the difference between the co-inoculations was insignificant. While MJ1 and DSM4166 could increase the HN content after reducing 30% of fertilization, the co-inoculation with DSM4166 or CHA0-*nif* after 30%-reducing fertilization showed no significant effects.

In the acid soil grown with lettuce, compared with the regular fertilization, the co-inoculation of MJ1 with CHA0-*nif* significantly improved OM, HN, TN, AP, and AK in the 0–20 cm of soil, while only the contents of HN and AP were elevated by MJ1 with DSM4166 and the promoted effect of CHA0-*nif* was stronger than that of DSM4166 in soil AP. After reducing 30% of fertilization, the combination of MJ1 and DSM4166 could improve soil SS, while MJ1 with CHA0-*nif* was found to improve soil AP. DSM4166 inoculating with MJ1 dramatically enhanced the HN and AP in 20–40 cm soil, while the soil treated with CHA0-*nif* with MJ1 possessed a higher content of HN, TN, and AK than DSM4166, *p* < 0.05. For the treatment with 30%-reducing fertilization reduction, the combination of DSM4166 or CHA0-*nif* with MJ1 could keep the soil content of TN, AP, and AK from reducing, and DSM4166 could improve HN and AK content.

### Effect of inoculated strains on crop quality

For the yield production of cucumber, all treatments showed significantly enhanced effect even the treatments with a 30% reducing regular fertilization, the treatment of MJ1 and CHA0-*nif* combination showed the maximum production of 5542.36 ± 14.60 kg/acre. For the content of protein and vitamin C in cucumber, MJ1 combined with DSM4166 or CHA0-*nif* showed a dramatically promoted effect in the presence of regular fertilization. Interestingly, after reducing 30% fertilization, the treatments of MJ1 and CHA0-*nif* combination showed the maximum contents of protein (0.737 ± 0.021%) and vitamin C (4.287 ± 0.032 mg/kg) in all treatments (Table 3).

**Table 3:** The quality indexes of cucumber and lettuce in different groups.

	Crops												
	Cucumber			Lettuce									
	Protein (%)	Vitamin C (mg/kg)	Yield production (kg/acre)	Head weight (kg)	Vanes width (cm)	Vanes length (cm)	Head height (cm)	Vitamin C (mg/kg)	Nitrate (mg/kg)	Soluble protein (mg/kg)	Soluble sugar (mg/kg)	Crude fiber (%)	Yield production (kg/acre)
C/L-1	0.487 ± 0.012d	2.057 ± 0.021e	3555.95 ± 13.97e	0.210 ± 0.027b	13.93 ± 0.50a	14.03 ± 0.57a	11.53 ± 0.50a	58.33 ± 2.54c	404.71 ± 13.42d	2842.42 ± 153.88d	8.10 ± 0.05a	1.217 ± 0.064c	1005.97 ± 134.03b
C/L-2	0.610 ± 0.010b	2.227 ± 0.012d	4679.59 ± 6.13b	0.240 ± 0.026ab	14.11 ± 1.12a	14.95 ± 1.41a	12.53 ± 1.54a	66.67 ± 2.30bc	536.53 ± 3.98a	2947.72 ± 73.06cd	8.80 ± 1.71a	1.663 ± 0.258bc	1200.00 ± 132.29ab
C/L-3	0.570 ± 0.026c	2.963 ± 0.045b	5542.36 ± 14.60a	0.269 ± 0.046a	14.41 ± 0.55a	14.71 ± 0.48a	12.23 ± 0.64a	83.33 ± 10.43a	555.37 ± 13.22a	3090.64 ± 1.96bc	9.72 ± 0.16a	2.207 ± 0.146ab	1342.60 ± 231.90a
C/L-4	0.617 ± 0.015b	2.337 ± 0.023c	4073.55 ± 73.01d	0.231 ± 0.017ab	14.01 ± 0.74a	14.09 ± 1.10a	11.61 ± 1.66a	58.33 ± 1.84c	480.88 ± 14.18c	3617.15 ± 135.61a	10.29 ± 2.09a	2.583 ± 0.947ab	1152.37 ± 84.62ab
C/L-5	0.737 ± 0.021a	4.287 ± 0.032a	4571.46 ± 44.84c	0.242 ± 0.036ab	14.07 ± 0.55a	14.53 ± 1.10a	11.90 ± 0.40a	75.66 ± 3.01ab	502.64 ± 1.61b	3233.55 ± 92.00b	10.60 ± 2.78a	2.980 ± 0.580a	1208.33 ± 180.42ab

The difference is marked with a letter. If the difference is not significant, the same letter a will be marked until the average number with significant difference is met, followed by the letter b, and so on. *p* < 0.05 indicates statistical significance.

The production of lettuce was increased by the combination of MJ1 and CHA0-*nif* under regular fertilization, and no significant difference was observed between different strain combinations. The quality of lettuce was assessed from two aspects, including growth indexes and physiological indexes. The lettuce that grew under the fertilization combined with MJ1 and CHA0-*nif* possessed the highest head weight of 0.269 ± 0.046 kg but was insignificantly different from other inoculated treatments. For the other growth indexes, no significant difference was observed in the lettuce vanes’ width, length, and the height of lettuce heads. Physiologically, the content of vitamin C, nitrate, soluble protein, and crude fiber of lettuce was significantly improved in the presence of MJ1 and CHA0-*nif*, while the combination of MJ1 with DSM4166 was only found to enhance the content of nitrate, which was insignificantly different from CHA0-*nif* (Table 3).

### Effect of inoculated strains on microbial biomass of barrier soil

In salinized soil, the co-inoculation of MJ1 with DSM4166 or CHA0-*nif* significantly increased the MBC and MBN in 0–20 cm depth and the MBN in 20–40 cm in the presence of regular fertilization, and there was no significant difference in the effect between DSM4166 and CHA0-*nif*. In the presence of 70% of regular fertilization, the combination of MJ1 and DSM4166 or CHA0-*nif* markedly improved the MBN of 20–40 cm soil, and their effects on the MBC, MBN, and MBP in 0–20 cm soil were not obvious (Table 4).

**Table 4:** Biomass soil grow with cucumber or lettuce in the presence of different inoculated strains.

		Cucumber			Lettuce		
		MBC (mg/kg)	MBN (mg/kg)	MBP (mg/kg)	MBC (mg/kg)	MBN (mg/kg)	MBP (mg/kg)
0–20 cm	C/L-1	418.33 ± 28.71b	46.91 ± 3.75c	46.58 ± 0.71a	345.38 ± 31.20b	46.76 ± 6.44a	51.36 ± 5.54b
	C/L-2	500.11 ± 36.67a	71.17 ± 6.91a	51.63 ± 5.66a	371.20 ± 21.09ab	47.88 ± 4.66a	62.85 ± 5.00a
	C/L-3	495.38 ± 11.50a	66.94 ± 5.86ab	49.84 ± 1.90a	394.56 ± 9.35a	50.84 ± 4.81a	58.27 ± 4.90ab
	C/L-4	426.91 ± 23.80b	55.75 ± 5.07c	46.94 ± 4.57a	369.96 ± 12.85ab	48.26 ± 5.50a	57.48 ± 6.05ab
	C/L-5	452.92 ± 24.76ab	57.80 ± 7.91bc	48.80 ± 5.18a	335.87 ± 25.07b	45.68 ± 4.88a	54.76 ± 6.93ab
20–40 cm	C/L-1	328.98 ± 34.04a	38.85 ± 4.86b	37.86 ± 2.34abc	287.65 ± 26.87b	36.03 ± 5.54b	46.14 ± 6.18ab
	C/L-2	367.43 ± 44.64a	47.91 ± 3.50a	41.72 ± 1.52a	310.35 ± 19.24b	33.49 ± 4.63b	51.18 ± 3.73a
	C/L-3	378.98 ± 42.39a	50.84 ± 4.81a	40.97 ± 0.72ab	378.98 ± 42.39a	48.71 ± 4.53a	40.97 ± 0.72b
	C/L-4	347.97 ± 42.84a	48.58 ± 4.12a	37.77 ± 1.53bc	310.53 ± 30.09b	36.29 ± 5.29b	47.89 ± 6.79ab
	C/L-5	331.04 ± 28.89a	49.62 ± 6.35a	34.81 ± 3.55c	322.45 ± 3.92b	35.47 ± 4.73b	53.93 ± 3.45a

The difference is marked with a letter. If the difference is not significant, the same letter a will be marked until the average number with significant difference is met, followed by the letter b, and so on. *p* < 0.05 indicates statistical significance.

According to the data, the co-inoculation of MJ1 with DSM4166 or CHA0-*nif* exerted an enhanced effect on the MBP level of 0–20 cm acid soil under regular fertilization. While only MJ1 inoculating with CHA0-*nif* was found to improve the level of MBC and MBN of 20–40 cm soil, the effect of DSM4166 was insignificant. After reducing regular fertilization, the combination of MJ1 with DSM4166 or CHA0-*nif* could protect the MBC, MBN, and MBP levels from declining (Table 4).

### Effect of inoculated strains on the alpha-diversity of barrier soil microbial community

Among the critical alpha-diversity indexes, there were no significant changes were observed in the bacterial richness and diversity between different groups of salinized soil (Supplementary Figure S1A). For the fungal community, the co-inoculation of MJ1 with DSM4166 or CHA0-*nif* was found to reduce the number of observed species and Shannon indexes, but the differences were not significant (Supplementary Figure S1B). Although there was no significant effect of inoculated strains was observed on the microbial diversity and richness, an increasing number of earthworms was found in the cucumber-grown soil (Supplementary Figure S2).

While in the acid soil, in the treatments with regular fertilization, the co-inoculation of MJ1 and DSM4166 or CHA0-*nif* significantly improved the number of bacterial species and the Shannon indexes, indicating the improving bacterial richness and diversity. After reducing 30% of regular fertilization, the combination of MJ1 and CHA0-*nif* showed a markedly promoted effect on the bacterial richness and diversity, but the effect of DSM4166 was insignificant (Supplementary Figure S1C). For the fungal community, the inoculation of MJ1 with DSM4166 or CHA0-*nif* slightly elevated the observed species number and Shannon index, but the changes were not of statistical significance (Supplementary Figure S1D).

### Effect of inoculated strains on the beta-diversity of barrier soil microbial community

**Figure 1:**
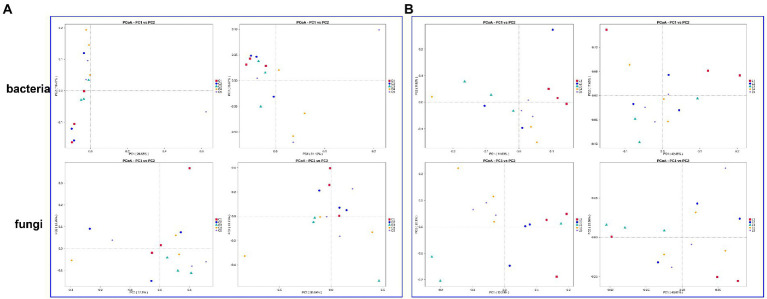
PCoA based on the weighted and unweighted unifrac distance algorithm. **(A,B)** The bacterial and fungal community of salinized **(A)** and acid **(B)** soil. The left rows of each part represented the results of unweighted unifrac distance and the right rows represented the results of the weighted unifrac distance algorithm.

Based on two distance algorithms, the microbial community structure of soil with different treatments were evaluated (Figure 1). In the bacterial community of salinized soil, the combination of MJ1 with DSM4166 or CHA0-*nif* under 30%-reducing regular fertilization was found to significantly separate from the regular fertilization group in the results of weighted unifrac (total explanation rate of 56.69%) and unweighted unifrac (total explanation rate of 37.05%; Figure 1A). The fungal community structure of the soil with the MJ1-CHA0-*nif* combination was found to separate from the regular fertilization from the results of weighted unifrac (total explanation rate of 48.85%) and unweighted unifrac (total explanation rate of 22.86%; Figure 1A).

While the bacterial and fungal community structure of the acid soil showed a stronger response to the inoculated strains. Specifically, both the weighted unifrac (total explanation rate of 60.53%) and unweighted unifrac (total explanation rate of 20.25%) demonstrated the clear separation between the regular fertilization treatment and the other treatments with different co-inoculations. The combination of MJ1 with CHA0-*nif* under regular fertilization and the combination of MJ1 with DSM4166 in the presence of 70% regular fertilization was found to be more different from the regular fertilization (Figure 1B). Meanwhile, a clear separation was also observed in the fungal community between the fertilization group and the various combinations of MJ1 and DSM4166 or CHA0-*nif* through the weighted unifrac (total explanation rate of 59.90%) and unweighted unifrac (total explanation rate of 23.69%). MJ1 combined with CHA0-*nif* was found to mostly affect the fungal community structure under regular or reduced fertilization (Figure 1B).

### The enriched differential microorganisms

In the acid soil, two bacterial and three fungal biomarkers were identified between different treatments. Moreover, in the presence of regular fertilization, five fungal biomarkers were screened under the combination of MJ1 with DSM4166 or CHA0-*nif*. After reducing 30% of fertilization, three bacterial biomarkers were screened (Supplementary Figures S3A, B). For different strain combinations, five bacterial biomarkers and six fungal biomarkers were identified in the presence of MJ1 with DSM4166, while seven fungal biomarkers were identified under the combination of MJ1 with CHA0-*nif*. No microbial biomarker was observed between DSM4166 and CHA0-*nif* in the presence of 70% fertilization with MJ1 (Supplementary Figures S4A, B).

**Figure 2:**
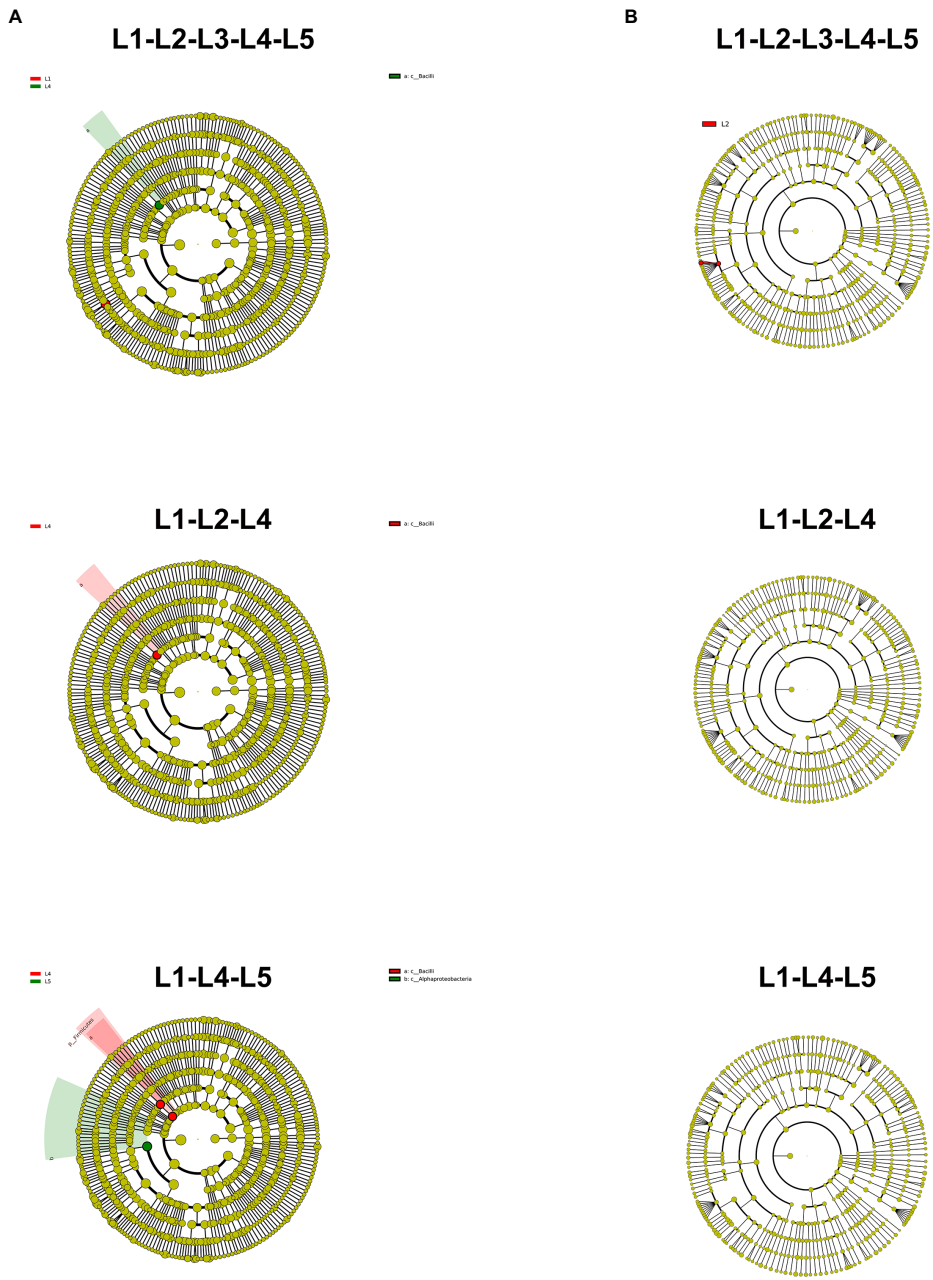
Lefse analysis to identify microbial biomarkers in each treatment of acid soil. **(A,B)** The results of bacterial **(A)** and fungal **(B)** communities. The yellow node represents that the species is not significantly enriched, and the nodes of other colors represent that the species are enriched in the corresponding treatment.

In the salinized soil, one bacterial biomarker were screened between different treatments. Additionally, in the presence of regular fertilization, one differential bacterium was identified, while two differential bacteria were observed after a 30% reduction of the fertilization (Figures 2A, B). One fungal biomarker was identified between DSM4166 and CHA0-*nif* in the presence of regular fertilization with MJ1, and one bacterial biomarker was screened after reducing 30% fertilization (Supplementary Figures S5A, B).

### Function prediction of enriched microorganisms

**Figure 3:**
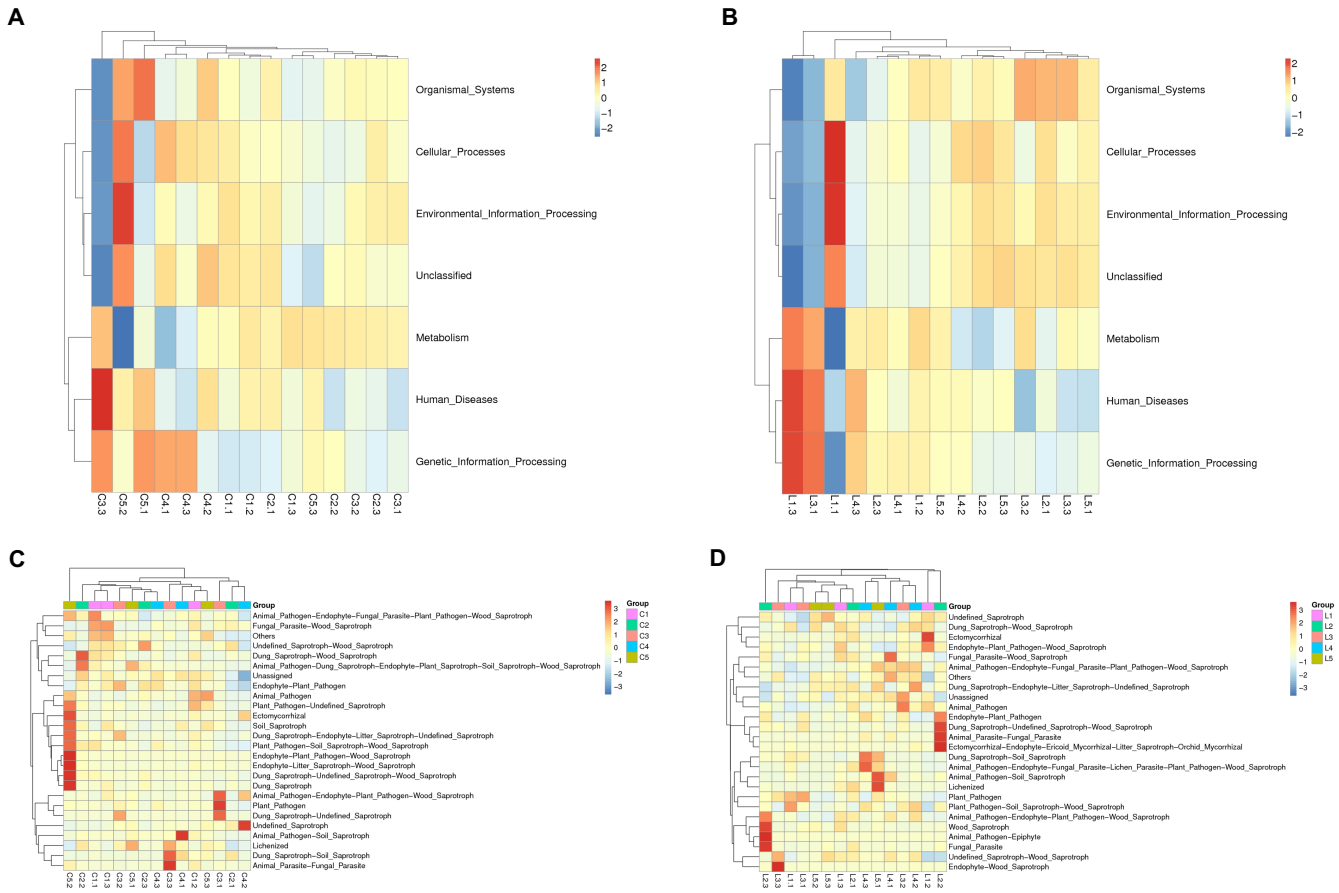
Heatmap of bacterial and fungal function prediction. **(A,B)** Function prediction of abundant bacteria in the salinized **(A)** and acid **(B)** soil. **(C,D)** Function prediction of abundant fungi in the salinized **(C)** and acid **(D)** soil.

The top 10 most abundant functions of enriched bacteria or fungi in the soil with different treatments were predicted and annotated. It was revealed that the functions of bacteria in both the salinized (Figure 3A) and acid (Figure 3B) soil were mainly associated with metabolism, genetic and environmental information processing, cellular processes, and a small part of human disease and organismal systems. The abundance of involved functions was relatively average between different treatments (Supplementary Figures S6A, B). The functions of the fungi community in both salinized (Figure 3C) and acid (Figure 3D) soil were mainly enriched as saprotrophs, especially the wood saprotroph-correlated function, such as plant and animal pathogen (Supplementary Figures S6C, D).

The PCA analysis on bacterial function enrichment of salinized soil showed that there was a clear separation of fertilization reduction treatments (MJ1 with DSM4166 or CHA0-*nif*) from the regular fertilization (Supplementary Figure S7A), while the difference in the fungal function was not obvious (Supplementary Figure S7B). Similarly in the acid soil, the bacterial functions of the reduction fertilization with MJ1 combined with DSM4166 or CHA0-*nif* were obviously separated from other groups (Supplementary Figure S7C). The fungal function under the regular fertilization with MJ1 combined with DSM4166 and the 30% reducing fertilization with MJ1 combined with CHA0-*nif* were separated from the other treatments (Supplementary Figure S7D).

### Association of soil physicochemical properties and microbial community

**Figure 4:**
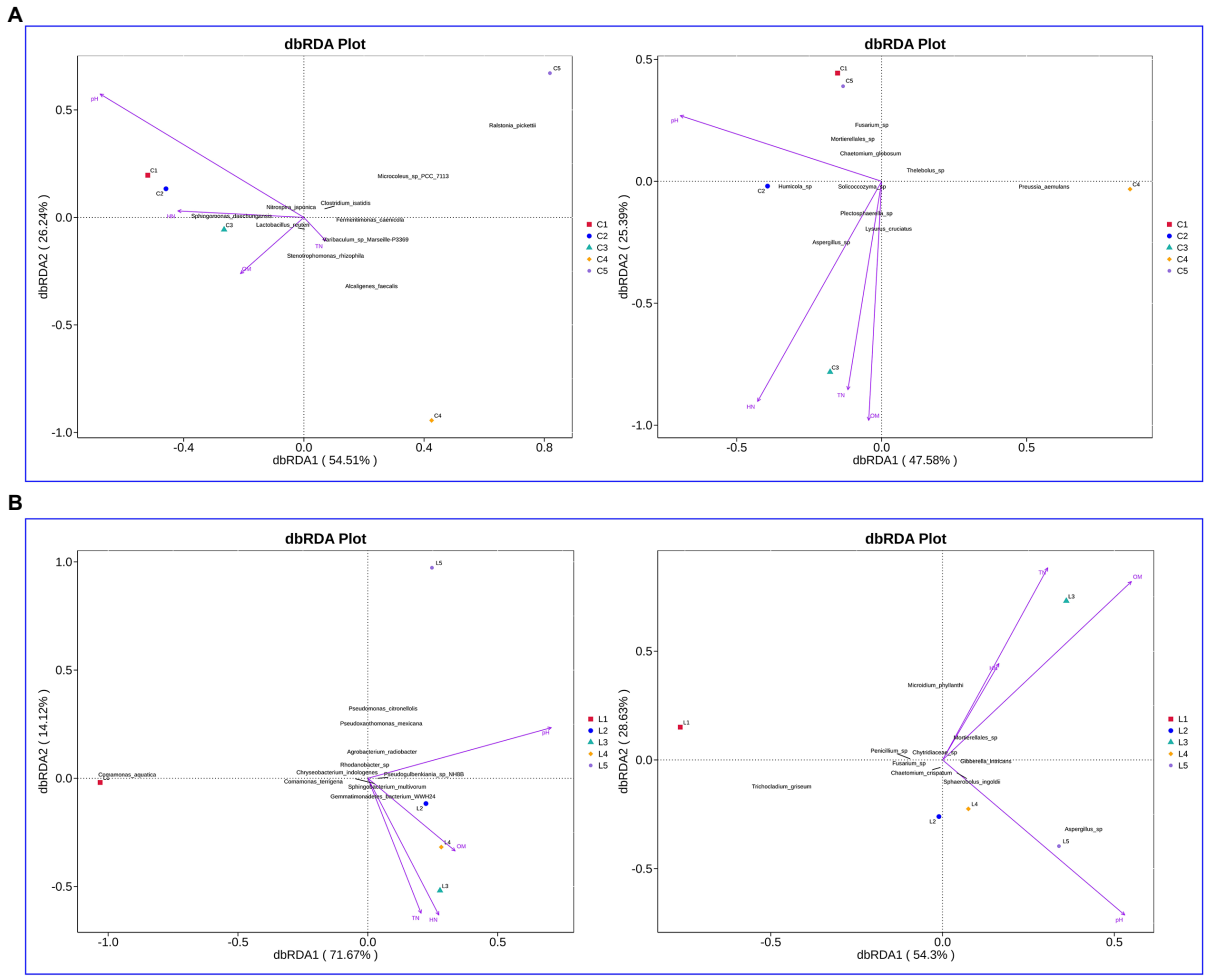
Evaluation of the association between soil physicochemical properties and microbial communities. **(A,B)** Effect of soil physicochemical properties on bacterial (left rows) and fungal (right rows) in the salinized **(A)** and acid **(B)** soil.

In the salinized soil, the interpretation rates of RDA1 for the bacterial and fungal communities were 54.51 and 47.58%, respectively, while the interpretation rates of RDA2 were 26.24 and 25.39%, respectively (Figure 4A). pH, HN, TN, and OM were demonstrated as the major affected factors associated with the bacterial and fungal community. The total interpretation rates of RDA were 85.79 and 82.93% for the bacterial and fungal communities in lettuce-grown soil, respectively (Figure 4B). The most correlated factors were also revealed as pH, HN, TN, and OM.

**Figure 5:**
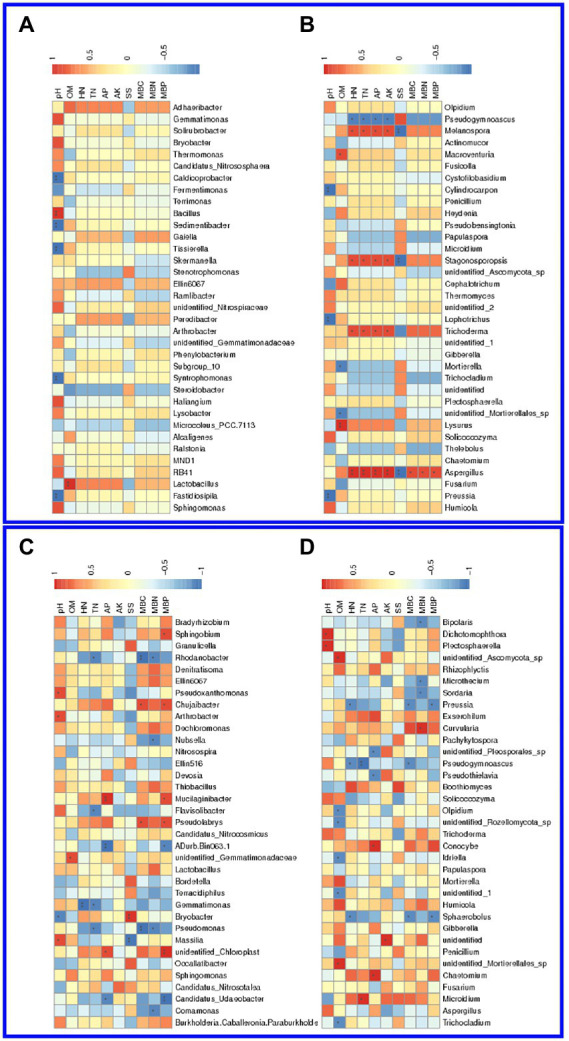
Association of soil physicochemical properties with microbial genera in the barrier soil. **(A,B)** The correlation of soil properties with bacteria **(A)** and fungi **(B)** genera in the salinized soil. **(C,D)** The correlation of soil properties with bacteria **(C)** and fungi **(D)** genera in the acid soil.

Using the CCA and RDA functions in the vegan package, we can calculate the *r*^2^ and *p* values of each environmental factor’s impact on species distribution through the envfit function, and then use the selected environmental factors with significant impact to do CCA and RDA analysis. In the salinized groups, pH was positively correlated with the *Bacillus* and negatively related to the *Caldicoprobacter*, *Sedimentibacter*, *Tissierella*, *Syntrophomonas*, and *Fastidiosipila* genus. The *Lactobacillus* was positively correlated with OM, while TN and HN showed no significant correlation with abundant bacterial genus (Figure 5A). pH showed a significantly negative correlation with the fungal genus, including *Cylindrocarpon*, *Lophotrichus*, and *Preussia*. The OM-correlated genus involved *Macroventuria*, *Lysurus*, and an unidentified *Mortierellales* genus. The correlation of TN or HN with fungal genera was consistent, where the *Pseudogymnoascus*, *Melanospora*, *Stagonosporopsis*, *Trichoderma*, and *Asepergillus* genera were all positively correlated with TN and HN (Figure 5B).

In the acid soil, *Pseudoxanthomonas*, *Arthrobacter*, and *Massilia* genus were positively correlated with soil pH, while the *Bryobacter* genus showed a significant negative correlation. OM was found to be positively correlated with an unidentified *Gemmatimonadaceae* genus, and HN was negatively correlated with *Gemmatimons* genus, which was also negatively correlated with TN as well the *Rhodanobacter*, *Flavisolibacter*, and *Pseudomonas* (Figure 5C). For the fungal genus, pH was found to be positively correlated with the *Dixhotomophthora* and *Plectosphaerella*, while OM showed significant correlation with a variety of genera, such as *Olpidium*, *ideriella*, *Trichocladium*, and several unidentified genera. Both TN and HN were negatively correlated with the Pseudogymnoascus genus, and TN also showed a positive correlation with the *Microidium* genus (Figure 5D).

## Discussion

### Effect of inoculated strains on barrier soil properties and crop quality

Due to the diversity of microbial function, the inoculated strains could ameliorate the soil environment and benefit the cycles of nutrients. For example, the inoculation of nitrogen-fixing microorganisms could fix the nitrogen elements in the air to meet the requirement of nitrogen and promote its uptake by plants (Reis and Teixeira, 2015; Dellagi et al., 2020; Aasfar et al., 2021). Phosphorus-solubilizing microorganisms could decompose a variety of organic acids, which can react with insoluble phosphates to convert them into available phosphorus (Alori et al., 2017). Consistently, although the inoculation of MJ1 with DSM4166 or CHA0-*nif* showed insignificant effect on soil pH, it dramatically elevated the contents of HN, TN, AP, and AK in shallow and deep soil layers.

Under regular fertilization, the combination of MJ1 with CHA0-*nif* was found to show a stronger enhancement than DSM4166. Additionally, after reducing 30% of regular fertilization the inoculated strains could keep or even elevated the nutrient contents in different soil layers, the difference in the effect of DSM4166 and CHA0-*nif* was not significant, indicating that the inoculation of MJ1 with DSM4166 or CHA0-*nif* could help reduce the application of regular fertilization and meet the requirement of carbon-neutral and environment friendly of the current status of agriculture. It is worthy to note that the effect of inoculated strains was distinct in different barrier soil. The combination of MJ1 with CHA0-*nif* was more effective to improve the content of nitrogen of salinized soil and the content of phosphorus in acid soil. Low availability of phosphorus is the typical barrier of acid soil due to the formation of precipitation with metal ions. Although there were no significant changes in pH of acid soil, the improved availability of soil nutrients also suggested the potential of inoculated strains in barrier soil remediation.

Microorganisms could produce a variety of metabolites, which could regulate plant growth and improve their ability to pest resistance. For example, it was reported that the inoculation of *Azospirillum brasilense* DSM-1843 could improve the content of chlorophyII and biomass, increase Fe in the cucumber leaves, and therefore promote the recovery of cucumbers from the Fe-deficiency (Pii et al., 2015). The application of microbiological fertilization significantly improved the content of nitrate and vitamin C in *Lactuca sativa* L. (Stojanovic et al., 2020). The production, protein content, and Vitamin C content of cucumber were promoted by the inoculated strains, meanwhile, the cucumber under 30%-reduction fertilization with MJ1 combining CHA0-*nif* showed the maximum contents of protein and Vitamin C. Therefore, this combination could benefit the target of “reducing fertilization and increasing production.” Although the effect of inoculated strains on lettuce production was not significant, the contents of Vitamin C, nitrate, soluble protein, and crude fiber of lettuce were dramatically improved by the combination of MJ1 with DSM4166 or CHA0-*nif*, especially the combination of MJ1 with CHA0-*nif* under regular or reducing fertilization. The increasing content of nitrate suggested that the fertilization can be reduced over 30% in the presence of inoculated strains, or the engineering bacteria can be established by knockout related regulator genes.

### Effect of inoculated strains on barrier soil microbial community

The secretion and metabolites of microorganisms play critical roles in affecting the microbial community (Tyc et al., 2017). Yuan et al. (2017) reported that *Bacillus amyloliquefaciens* NJN-6 released volatile organic compounds that dramatically affected soil bacterial and fungal communities and decreased the alpha-diversity of soil microbial community. The exogenous strains have also been revealed to affect the microbial community. For instance, in a cucumber-planting soil, the *myxobacterium Corallococcus* sp. strain EGB could regulate soil microbial community and further control cucumber Fusarium wilt (Ye et al., 2020). The application of the *Trichoderma hamatum* strain MHT1134 in the soil with continuous cropping obstacles could alleviate the reducing microbial diversity and abundance and improve microbial community structure (Mao and Jiang, 2021).

The application of fertilization could also affect the soil microbial community, and the fertilization amount is a critical factor associated with its effect. A recent study demonstrated that the long-term application of chemical fertilization would dramatically destroy the soil microbial community, which resulted in reduced bacterial diversity and increased soil healthy problems (Xu et al., 2020). Herein, the inoculation of MJ1 with DSM4166 or CHA0-nif was found to improve soil microbial biomass C, N, and P, especially in the deeper soil layers. It was observed that the reduction of regular fertilization did not affect the bacterial and fungal diversity of soil grown with cucumber. The inoculation of MJ1 with DSM4166 or CHA0-*nif* significantly affected the bacterial community of acid soil, where the inoculated strains improved the richness and diversity of soil bacteria. Although the effects were not significant, the inoculation of MJ1 with DSM4166 or CHA0-*nif* could also slightly improve fungal diversity and richness of acid soil. Additionally, the protective effect of inoculated strains on soil ecology was revealed in the salinized soil, where an increasing number of earthworms was observed even reducing the application of fertilization. The inoculated strains could keep or improved the microbial diversity after reducing fertilization application.

The weighted and unweighted unifrac distances were employed in the present study to evaluate the microbial community structure of the soil with different treatments, which could represent the kinship and the abundance of different species (Lozupone and Knight, 2005). The effect of exogenous strains on soil microbial beta-diversity has been previously reported. For example, *Rhizobium alamii* was revealed to improve significantly modified the beta-diversity of water stress soil and improved the tolerance of rapeseed (Tulumello et al., 2021). The inoculation of *Pigmentiphaga* sp. D-2 dramatically affected the bacterial community structure of an acetamiprid-contaminated area and improved the activity of acetamiprid degradation-related bacteria (Yang et al., 2021). The inoculated trains dramatically changed the microbial community structure of salinized soil, especially in the presence of 30%-reducing fertilization. Similarly, in acid soil, the response of bacterial and fungal community structure to inoculated strains was stronger than the cucumber-grown soil. Both the weighted unifrac and unweighted unifrac results showed significant changes in the bacterial and fungal community structure of lettuce-grown soil, indicating that the inoculation of MJ1 with DSM4166 or CHA0-*nif* could affect the relationship and abundance of bacteria and fungi.

Interestingly, except for the effect of inoculated strains, it was found that the reduction of regular fertilization significantly influenced the structure of the microbial community in cucumber-grown and lettuce-grown soil. Previously, the application of nitrogen fertilization was illustrated to drive the beta-diversity of soil archaea, bacteria, and fungi, and affected the microbial abundance (Li et al., 2020). The bacterial and fungal beta diversity in the soil was also found to be influenced by the application of biofertilizer (Yang et al., 2022). Therefore, the changes in the beta-diversity of soil bacteria and fungi after reducing fertilization also indicated the response of soil bacteria and fungi to fertilization, and the differential microorganisms might help explain the mechanism.

### Potential functions of enriched microorganisms

According to the Lefse analysis, several microbial biomarkers were identified in different treatments. *Pyrinomonadaceae* and *Blastocatellia* were enriched in the combination of MJ1 with DSM4166 in salinized soil. A previous study observed the relatively high abundance of these two genera, and *Blastocatellia* was demonstrated to be sensitive to soil pH, carbon, nitrogen, and drought (Wust et al., 2016; Ivanova et al., 2020; Huber et al., 2022). The enriched fungi were *Motiereallaes* and *Mortierellomycetes*, which were reported to be enriched in the shallow soil layers and negatively correlated with nitrogen in the soil (Zhou et al., 2021; Wang et al., 2022). For the different combinations of MJ1 with DSM4166 or CHA0-*nif*, *Mortierellomycetes* and *plectosphaerellaceae* were found to play vital roles in the inoculation of CHA0-*nif* and DSM4166, respectively, in the presence of regular fertilization. Interestingly, the identified biomarker microorganisms were significantly different between DSM4166 and CHA0-*nif* combined with MJ1 after reducing fertilization, indicating the different mechanism of the combinations of MJ1 with DSM4166 or CHA0-*nif* to meet the nutrients requirements of cucumber after reducing fertilization.

In the acid soil, *Bacilli* was identified as a major biomarker of different treatments, which plays a critical role in the combination of MJ1 with DSM4166 in the presence of regular fertilization or 30%-reducing fertilization. *Bacilli* have been widely applied as a nitrogen-fixing engineering bacteria and biopesticides due to its outstanding ability to fix nitrogen and antibacterial (Perez-Garcia et al., 2011). While *Sordariales* and *Burkholderiales* were enriched in the combination of MJ1 with DSM4166 under regular fertilization and 30%-reducing fertilization, respectively. Although few studies have reported the function of *Sordariales* and *Burkholderiales* in soil microbial communities, the function of enriched microorganisms was evaluated in the present study. Similar functions of enriched microorganisms were observed between salinized and acid soil, which were mainly associated with metabolism, genetic information processing, cellular processes, and human disease, and fungi were mainly related to the saprotroph process. The inoculated strains MJ1, DSM4166, and CHA0-*nif* were able to solubilize phosphorus and fix nitrogen. The different functional role of inoculated strains was considered the major reason for different enriched microorganism. Moreover, the regulatory effects of inoculate strains on correlated genes or procedures also contributed to the response of various functional microorganisms to the inoculations.

### Effect of soil properties on soil microbial community

A recent investigation focused on the effect of fertilization patterns on soil microbial community revealed that the fertilization patterns could affect the nutrient status, which further affected the soil microbial biomass and microbial community structure (Li et al., 2022). In the present study, pH, OM, HN, and TN were identified to be the major factors correlated with the soil microbial community, which were found to strongly respond to the inoculated strains.

Although no significant changes were observed in the presence of different inoculated strains, pH was revealed to be the major effect factor in the salinized soil bacterial community, which correlated with several genera, including *Bacillus*. *Bacillus* was previously demonstrated to be alkaliphilic and possess the ability to solubilize phosphorus (Krulwich et al., 1999; Saeid et al., 2018). In terms of the fugal community, *Aspergillus*, *Pseudogymnoascus*, *Melanospora*, *Stagonosporopsis*, and *Trichoderma* were found to be more sensitive to soil properties, which were correlated with HN, TN, AP, AK, and SS. *Aspergillus* was also found to respond to soil biomass C, N, and P. These identified fungi have also been reported to play various functions in plant growth, including serving as pathogens and promoting crop production (Marin-Felix et al., 2018; Dou et al., 2019; Dong et al., 2021; Urbina et al., 2021). In the inoculated strains, MJ1 is a typical phosphorus-solubilizing bacteria, and the other strains DSM4166 and CHA0-*nif* were able to fix nitrogen, which improves the nutrient provision for the parasitic fungi. The acid soil properties-correlated bacterial genus was reported to play role in the rhizosphere, and the fungal genus was mainly involved in the plant diseases (Cho et al., 2017; Huq, 2019; Lee et al., 2019; Al-Sadi, 2021), which are consistent with the results of function prediction. Previously, Ren et al. (2021) tried to use organic fertilizer to replace chemical fertilizer and found that the effect of organic fertilizer on soil physicochemical properties influenced the microbial community and directly or indirectly affected the activity of soil enzymes. Therefore, it was speculated that the inoculated strains might affect the microbial community *via* regulating soil physicochemical properties.

### Outlook

This study focused on the growth and development of cucumber planting on salinized soil and lettuce on acid soil, which lacks negative controls. There have been some intolerant plants of salinized, acid, or other barrier soils identified in previous studies. Therefore, investigating the optimized fertilization or other management strategies would be beneficial and of urgent needs to improve the growth and development of sensitive plants on barrier soil.

## Conclusion

The cucumber (yield production, protein, and vitamin C) and lettuce quality indexes (yield production, vitamin C, nitrate, soluble protein, and crude fiber) and soil properties (OM, HN, TN, AP, AK, and SS) showed a significant response to the inoculation of phosphorus-solubilizing bacterium MJ1 with nitrogen-fixing bacteria DSM4166 and CHA0-*nif*. The combination of MJ1 with DSM4166 or CHA0-nif influenced the diversity and richness of bacterial community in the acid soil and showed no significant effects on the salinized soil microbial community and the fungal community of acid soil. The organismal system-, cellular process-, and metabolism-correlated bacteria and saprophytic fungi were enriched, which were speculated to mediate the response to inoculated strains. pH, OM, HN, and TN were identified to be the major factors correlated with the soil microbial community. The inoculation of MJ1 with DSM4166 and CHA0-*nif* could meet the requirement of cucumber and lettuce growth after reducing fertilization, which provides a novel candidate for the eco-friendly technique to meet the carbon-neutral topic.

## Data availability statement

The datasets presented in this study can be found in online repositories. The names of the repository/repositories and accession number(s) can be found in the article/Supplementary material.

## Author contributions

HN, YZ, and QT: conceptualization. HN and YW: methodology. HN, RZ, SR, and ZQ: software. HN, YW, RZ, and DP: validation. HN, LY, and JL: formal analysis. HN, YW, QW, GZ, LL, TL, and QT: investigation. TL, YZ, and QT: resources. HN: data curation and visualization. HN and QT: writing—original draft preparation. YZ and QT: writing—review and editing, supervision, and funding acquisition. All authors contributed to the article and approved the submitted version.

## Funding

This research was supported by funding from the National Key R&D Program of China (Grant No. 2019YFA0904000); the Recruitment Program of Global Experts (1000 Plan); Shandong Key Research and Development Program (2019JZZY010724); Qingdao Science and Technology Benefit People (Grant no. 21-1-4-ny-22-nsh); Natural Science Foundation of Shandong Province (ZR2021QC170), and the Program of Introducing Talents of Discipline to Universities (B16030).

## Conflict of interest

HN, RZ, SR, ZQ, and LL are employed by Qingdao Hexie Biotechnology Co., Ltd.

The remaining authors declare that the research was conducted in the absence of any commercial or financial relationships that could be construed as a potential conflict of interest.

## Publisher’s note

All claims expressed in this article are solely those of the authors and do not necessarily represent those of their affiliated organizations, or those of the publisher, the editors and the reviewers. Any product that may be evaluated in this article, or claim that may be made by its manufacturer, is not guaranteed or endorsed by the publisher.

## References

[ref1] Aasfar A. Bargaz A. Yaakoubi K. Hilali A. Bennis I. Zeroual Y.. (2021). Nitrogen fixing *Azotobacter* species as potential soil biological enhancers for crop nutrition and yield stability. Front. Microbiol. 12:628379. doi: 10.3389/fmicb.2021.628379, PMID: 33717018 PMC7947814

[ref2] Alori E. T. Glick B. R. Babalola O. O. (2017). Microbial phosphorus solubilization and its potential for use in sustainable agriculture. Front. Microbiol. 8:971. doi: 10.3389/fmicb.2017.00971, PMID: 28626450 PMC5454063

[ref3] Al-Sadi A. M. (2021). *Bipolaris sorokiniana*-induced black point, common root rot, and spot blotch diseases of wheat: a review. Front. Cell. Infect. Microbiol. 11:584899. doi: 10.3389/fcimb.2021.584899, PMID: 33777829 PMC7991903

[ref4] Bai Y. C. Chang Y. Y. Hussain M. Lu B. Zhang J. P. Song X. B.. (2020). Soil chemical and microbiological properties are changed by Long-term chemical fertilizers that limit ecosystem functioning. Microorganisms 8:694. doi: 10.3390/microorganisms8050694, PMID: 32397341 PMC7285516

[ref5] Balkhair K. S. (2016). Microbial contamination of vegetable crop and soil profile in arid regions under controlled application of domestic wastewater. Saudi J. Biol. Sci. 23, S83–S92. doi: 10.1016/j.sjbs.2015.10.029, PMID: 26858571 PMC4705318

[ref6] Cho G. Y. Lee J. C. Whang K. S. (2017). *Rhodanobacter rhizosphaerae* sp. nov., isolated from soil of ginseng rhizosphere. Int. J. Syst. Evol. Microbiol. 67, 1387–1392. doi: 10.1099/ijsem.0.001825, PMID: 28126050

[ref7] Curatti L. Rubio L. M. (2014). Challenges to develop nitrogen-fixing cereals by direct nif-gene transfer. Plant Sci. 225, 130–137. doi: 10.1016/j.plantsci.2014.06.003, PMID: 25017168

[ref8] Dellagi A. Quillere I. Hirel B. (2020). Beneficial soil-borne bacteria and fungi: a promising way to improve plant nitrogen acquisition. J. Exp. Bot. 71, 4469–4479. doi: 10.1093/jxb/eraa112, PMID: 32157312 PMC7475097

[ref9] do Carmo Dias B. da Mota F. F. Jurelevicius D. Seldin L. (2021). Genetics and regulation of nitrogen fixation in *Paenibacillus brasilensis* PB24. Microbiol. Res. 243:126647. doi: 10.1016/j.micres.2020.126647, PMID: 33290933

[ref10] Dong Z. Y. Huang Y. H. Manawasinghe I. S. Wanasinghe D. N. Liu J. W. Shu Y. X.. (2021). *Stagonosporopsis pogostemonis*: a novel ascomycete fungus causing leaf spot and stem blight on *Pogostemon cablin* (Lamiaceae) in South China. Pathogens 10:1093. doi: 10.3390/pathogens10091093, PMID: 34578126 PMC8465882

[ref11] Dou K. Gao J. Zhang C. Yang H. Jiang X. Li J.. (2019). Trichoderma biodiversity in major ecological systems of China. J. Microbiol. 57, 668–675. doi: 10.1007/s12275-019-8357-7, PMID: 31124048

[ref01] Edgar R. C. Haas B. J. Clemente J. C. Quince C. Knight R. (2011). UCHIME improves sensitivity and speed of chimera detection. Bioinformatics (Oxford, England) 27, 2194–2200. doi: 10.1111/1751-7915.13335, PMID: 21700674 PMC3150044

[ref12] Huber K. J. Vieira S. Sikorski J. Wüst P. K. Fösel B. U. Gröngröft A.. (2022). Differential response of acidobacteria to water content, soil type, and land use during an extended drought in African Savannah soils. Front. Microbiol. 13:750456. doi: 10.3389/fmicb.2022.750456, PMID: 35222321 PMC8874233

[ref13] Huq M. A. (2019). *Sphingobium chungangianum* sp. nov., isolated from rhizosphere of *Pinus koraiensis*. Antonie Van Leeuwenhoek 112, 1341–1348. doi: 10.1007/s10482-019-01266-8, PMID: 30997587

[ref14] Ivanova A. A. Zhelezova A. D. Chernov T. I. Dedysh S. N. (2020). Linking ecology and systematics of acidobacteria: distinct habitat preferences of the acidobacteria and *Blastocatellia* in tundra soils. PLoS One 15:e0230157. doi: 10.1371/journal.pone.0230157, PMID: 32182280 PMC7077872

[ref15] Jing X. Cui Q. Li X. Yin J. Ravichandran V. Pan D.. (2020). Engineering pseudomonas protegens Pf-5 to improve its antifungal activity and nitrogen fixation. Microb. Biotechnol. 13, 118–133. doi: 10.1111/1751-7915.13335, PMID: 30461205 PMC6984399

[ref16] Klaic R. Guimarães G. G. F. Giroto A. S. Bernardi A. C. C. Zangirolami T. C. Ribeiro C.. (2021). Synergy of *Aspergillus niger* and components in biofertilizer composites increases the availability of nutrients to plants. Curr. Microbiol. 78, 1529–1542. doi: 10.1007/s00284-021-02406-y, PMID: 33675402

[ref17] Krulwich T. A. Guffanti A. A. Ito M. (1999). pH tolerance in bacillus: alkaliphiles versus non-alkaliphiles. Novartis Found. Symp. 221, 167–179. doi: 10.1002/9780470515631.ch11, PMID: 10207919

[ref18] Lee J. C. Song J. S. Whang K. S. (2019). *Sphingobium pinisoli* sp. nov., isolated from the rhizosphere soil of a Korean native pine tree. Antonie Van Leeuwenhoek 112, 815–825. doi: 10.1007/s10482-018-01215-x, PMID: 30565024

[ref19] Li H. Liu J. Li G. Shen J. Bergstrom L. Zhang F. (2015). Past, present, and future use of phosphorus in Chinese agriculture and its influence on phosphorus losses. Ambio 44, 274–285. doi: 10.1007/s13280-015-0633-0, PMID: 25681984 PMC4329154

[ref20] Li Y. Tremblay J. Bainard L. D. Cade-Menun B. Hamel C. (2020). Long-term effects of nitrogen and phosphorus fertilization on soil microbial community structure and function under continuous wheat production. Environ. Microbiol. 22, 1066–1088. doi: 10.1111/1462-2920.14824, PMID: 31600863

[ref21] Li L. Xiang D. Wu Y. F. Huang Y. D. Li H. Zhang X. M.. (2022). Effects of long-term different fertilization patterns on soil nutrients and microbial community structure of tomato in a solar greenhouse. Ying Yong Sheng Tai Xue Bao 33, 415–422. doi: 10.13287/j.1001-9332.202202.027, PMID: 35229515

[ref22] Lozupone C. Knight R. (2005). UniFrac: a new phylogenetic method for comparing microbial communities. Appl. Environ. Microbiol. 71, 8228–8235. doi: 10.1128/AEM.71.12.8228-8235.2005, PMID: 16332807 PMC1317376

[ref23] Mahmud K. Makaju S. Ibrahim R. Missaoui A. (2020). Current Progress in nitrogen fixing plants and microbiome research. Plants 9:97. doi: 10.3390/plants9010097, PMID: 31940996 PMC7020401

[ref24] Mao T. Jiang X. (2021). Changes in microbial community and enzyme activity in soil under continuous pepper cropping in response to *Trichoderma hamatum* MHT1134 application. Sci. Rep. 11:21585. doi: 10.1038/s41598-021-00951-x, PMID: 34732764 PMC8566488

[ref25] Marin-Felix Y. Guarro J. Ano-Lira J. F. Garcia D. Iller A. N. Stchigel A. M. (2018). *Melanospora* (Sordariomycetes, Ascomycota) and its relatives. MycoKeys 44, 81–122. doi: 10.3897/mycokeys.44.29742, PMID: 30598621 PMC6306512

[ref26] Parro V. Moreno-Paz M. (2004). Nitrogen fixation in acidophile iron-oxidizing bacteria: the nif regulon of *Leptospirillum ferrooxidans*. Res. Microbiol. 155, 703–709. doi: 10.1016/j.resmic.2004.05.010, PMID: 15501646

[ref27] Perez-Garcia A. Romero D. de Vicente A. (2011). Plant protection and growth stimulation by microorganisms: biotechnological applications of bacilli in agriculture. Curr. Opin. Biotechnol. 22, 187–193. doi: 10.1016/j.copbio.2010.12.003, PMID: 21211960

[ref28] Pii Y. Penn A. Terzano R. Crecchio C. Mimmo T. Cesco S. (2015). Plant-microorganism-soil interactions influence the Fe availability in the rhizosphere of cucumber plants. Plant Physiol. Biochem. 87, 45–52. doi: 10.1016/j.plaphy.2014.12.014, PMID: 25544744

[ref29] Rawat P. Das S. Shankhdhar D. Shankhdhar S. C. (2021). Phosphate-solubilizing microorganisms: mechanism and their role in phosphate solubilization and uptake. J. Soil Sci. Plant Nutr. 21, 49–68. doi: 10.1007/s42729-020-00342-7

[ref30] Reis V. M. Teixeira K. R. (2015). Nitrogen fixing bacteria in the family Acetobacteraceae and their role in agriculture. J. Basic Microbiol. 55, 931–949. doi: 10.1002/jobm.201400898, PMID: 25736602 PMC7166518

[ref31] Ren J. Liu X. Yang W. Yang X. Li W. Xia Q.. (2021). Rhizosphere soil properties, microbial community, and enzyme activities: short-term responses to partial substitution of chemical fertilizer with organic manure. J. Environ. Manag. 299:113650. doi: 10.1016/j.jenvman.2021.113650, PMID: 34481370

[ref32] Saeid A. Prochownik E. Dobrowolska-Iwanek J. (2018). Phosphorus solubilization by *Bacillus* species. Molecules 23. doi: 10.3390/molecules23112897, PMID: 30404208 PMC6278551

[ref33] Sánchez-Rangel J. C. Benavides J. Heredia J. B. Cisneros-Zevallos L. Jacobo-Velázquez D. A. (2013). The Folin-Ciocalteu assay revisited: improvement of its specificity for total phenolic content determination. Anal. Methods 5, 5990–5999. doi: 10.1039/c3ay41125g

[ref34] Schneider K. D. van Straaten P. de Orduña R. M. Glasauer S. Trevors J. Fallow D.. (2010). Comparing phosphorus mobilization strategies using *Aspergillus niger* for the mineral dissolution of three phosphate rocks. J. Appl. Microbiol. 108, 366–374. doi: 10.1111/j.1365-2672.2009.04489.x, PMID: 19709342

[ref35] Stojanovic M. Petrovic I. Zuza M. Jovanovic Z. Moravcevic D. Cvijanovic G.. (2020). The productivity and quality of *Lactuca sativa* as influenced by microbiological fertilisers and seasonal conditions. Zemdirbyste-Agriculture 107, 345–352. doi: 10.13080/z-a.2020.107.044

[ref36] Thiel T. (2019). Organization and regulation of cyanobacterial nif gene clusters: implications for nitrogenase expression in plant cells. FEMS Microbiol. Lett. 366:fnz077. doi: 10.1093/femsle/fnz077, PMID: 31062027

[ref37] Tulumello J. Chabert N. Rodriguez J. Long J. Nalin R. Achouak W.. (2021). Rhizobium alamii improves water stress tolerance in a non-legume. Sci. Total Environ. 797:148895. doi: 10.1016/j.scitotenv.2021.148895, PMID: 34346368

[ref38] Tyc O. Song C. Dickschat J. S. Vos M. Garbeva P. (2017). The ecological role of volatile and soluble secondary metabolites produced by soil bacteria. Trends Microbiol. 25, 280–292. doi: 10.1016/j.tim.2016.12.002, PMID: 28038926

[ref39] Urbina J. Chestnut T. Allen J. M. Levi T. (2021). *Pseudogymnoascus* destructans growth in wood, soil and guano substrates. Sci. Rep. 11:763. doi: 10.1038/s41598-020-80707-1, PMID: 33436940 PMC7804951

[ref40] Wang Y. Y. Cheng Y. H. Chen K. E. Tsay Y. F. (2018). Nitrate transport, signaling, and use efficiency. Annu. Rev. Plant Biol. 69, 85–122. doi: 10.1146/annurev-arplant-042817-040056 29570365

[ref41] Wang J. Liao L. Wang G. Liu H. Wu Y. Liu G.. (2022). N-induced root exudates mediate the rhizosphere fungal assembly and affect species coexistence. Sci. Total Environ. 804:150148. doi: 10.1016/j.scitotenv.2021.150148, PMID: 34520919

[ref42] Wu W. L. Yan J. L. Jiang T. Wei S. Q. (2019). Natural organic matter-metal ion/oxide-phosphorus complexes in environment: a review. J. Ecol. Rural Environ. 35, 1089–1096. doi: 10.19741/j.issn.1673-4831.2018.0636

[ref43] Wust P. K. Foesel B. U. Geppert A. Huber K. J. Luckner M. Wanner G.. (2016). *Brevitalea aridisoli*, *B. deliciosa* and *Arenimicrobium luteum*, three novel species of Acidobacteria subdivision 4 (class Blastocatellia) isolated from savanna soil and description of the novel family Pyrinomonadaceae. Int. J. Syst. Evol. Microbiol. 66, 3355–3366. doi: 10.1099/ijsem.0.001199, PMID: 27255677

[ref44] Xiao C. Wu X. Chi R. (2015). Dephosphorization of high-phosphorus iron ore using different sources of *Aspergillus niger* strains. Appl. Biochem. Biotechnol. 176, 518–528. doi: 10.1007/s12010-015-1592-4, PMID: 25822597

[ref45] Xu G. Fan X. Miller A. J. (2012). Plant nitrogen assimilation and use efficiency. Annu. Rev. Plant Biol. 63, 153–182. doi: 10.1146/annurev-arplant-042811-105532 22224450

[ref46] Xu Q. Ling N. Chen H. Duan Y. Wang S. Shen Q.. (2020). Long-term chemical-only fertilization induces a diversity decline and deep selection on the soil bacteria. mSystems 5:e00337–20. doi: 10.1128/mSystems.00337-20 32665327 PMC7363003

[ref47] Yan P. Wu L. Wang D. Fu J. Shen C. Li X.. (2020). Soil acidification in Chinese tea plantations. Sci. Total Environ. 715:136963. doi: 10.1016/j.scitotenv.2020.136963, PMID: 32014781

[ref48] Yang H. Zhang Y. Chuang S. Cao W. Ruan Z. Xu X.. (2021). Bioaugmentation of acetamiprid-contaminated soil with *Pigmentiphaga* sp. strain D-2 and its effect on the soil microbial community. Ecotoxicology 30, 1559–1571. doi: 10.1007/s10646-020-02336-8, PMID: 33443714

[ref49] Yang L. Y. Zhou S. Y. Lin C. S. Huang X. R. Neilson R. Yang X. R. (2022). Effects of biofertilizer on soil microbial diversity and antibiotic resistance genes. Sci. Total Environ. 820:153170. doi: 10.1016/j.scitotenv.2022.153170, PMID: 35051473

[ref50] Ye X. Li Z. Luo X. Wang W. Li Y. Li R.. (2020). A predatory myxobacterium controls cucumber Fusarium wilt by regulating the soil microbial community. Microbiome 8:49. doi: 10.1186/s40168-020-00824-x, PMID: 32252828 PMC7137222

[ref51] Yu F. Jing X. Li X. Wang H. Chen H. Zhong L.. (2019). Recombineering pseudomonas protegens CHA0: an innovative approach that improves nitrogen fixation with impressive bactericidal potency. Microbiol. Res. 218, 58–65. doi: 10.1016/j.micres.2018.09.009, PMID: 30454659

[ref52] Yu H. Yuan M. Lu W. Yang J. Dai S. Li Q.. (2011). Complete genome sequence of the nitrogen-fixing and rhizosphere-associated bacterium pseudomonas stutzeri strain DSM4166. J. Bacteriol. 193, 3422–3423. doi: 10.1128/JB.05039-11, PMID: 21515765 PMC3133286

[ref53] Yuan J. Zhao M. Li R. Huang Q. Raza W. Rensing C.. (2017). Microbial volatile compounds alter the soil microbial community. Environ. Sci. Pollut. Res. Int. 24, 22485–22493. doi: 10.1007/s11356-017-9839-y, PMID: 28803260

[ref54] Zhang M. Muhammad R. Zhang L. Xia H. Cong M. Jiang C. (2019). Investigating the effect of biochar and fertilizer on the composition and function of bacteria in red soil. Appl. Soil Ecol. 139, 107–116. doi: 10.1016/j.apsoil.2019.03.021

[ref55] Zhang M. Zhang L. Riaz M. Xia H. Jiang C. (2021). Biochar amendment improved fruit quality and soil properties and microbial communities at different depths in citrus production. J. Clean. Prod. 292:126062. doi: 10.1016/j.jclepro.2021.126062

[ref56] Zhou J. B. Jin Z. J. Xiao X. Y. Leng M. Wang X. T. Pan F. J. (2021). Investigation of soil fungal communities and functionalities within karst Paddy fields. Huan Jing Ke Xue 42, 4005–4014. doi: 10.13227/j.hjkx.202011164, PMID: 34309287

[ref57] Zhu Q. Liu X. Hao T. Zeng M. Shen J. Zhang F.. (2018). Modeling soil acidification in typical Chinese cropping systems. Sci. Total Environ. 613–614, 1339–1348. doi: 10.1016/j.scitotenv.2017.06.257, PMID: 28968946

