# Supplementary material for: Combination of Aspergillus niger MJ1 with Pseudomonas stutzeri DSM4166 or mutant Pseudomonas fluorescens CHA0-nif improved crop quality, soil properties, and microbial communities in barrier soil
Source: Front Microbiol. 2023 Feb 2;14:1064358. doi: 10.3389/fmicb.2023.1064358 (PMC9932699; doi:10.3389/fmicb.2023.1064358)
Supplement: Supplementary file 1 [file Data_Sheet_1.docx]

**Supplementary materials**

Combination of *Aspergillus niger* MJ1 with *Pseudomonas stutzeri* DSM4166 or mutant *Pseudomonas fluorescens* CHA0-*nif* improved crop quality, soil properties, and microbial communities in barrier soil


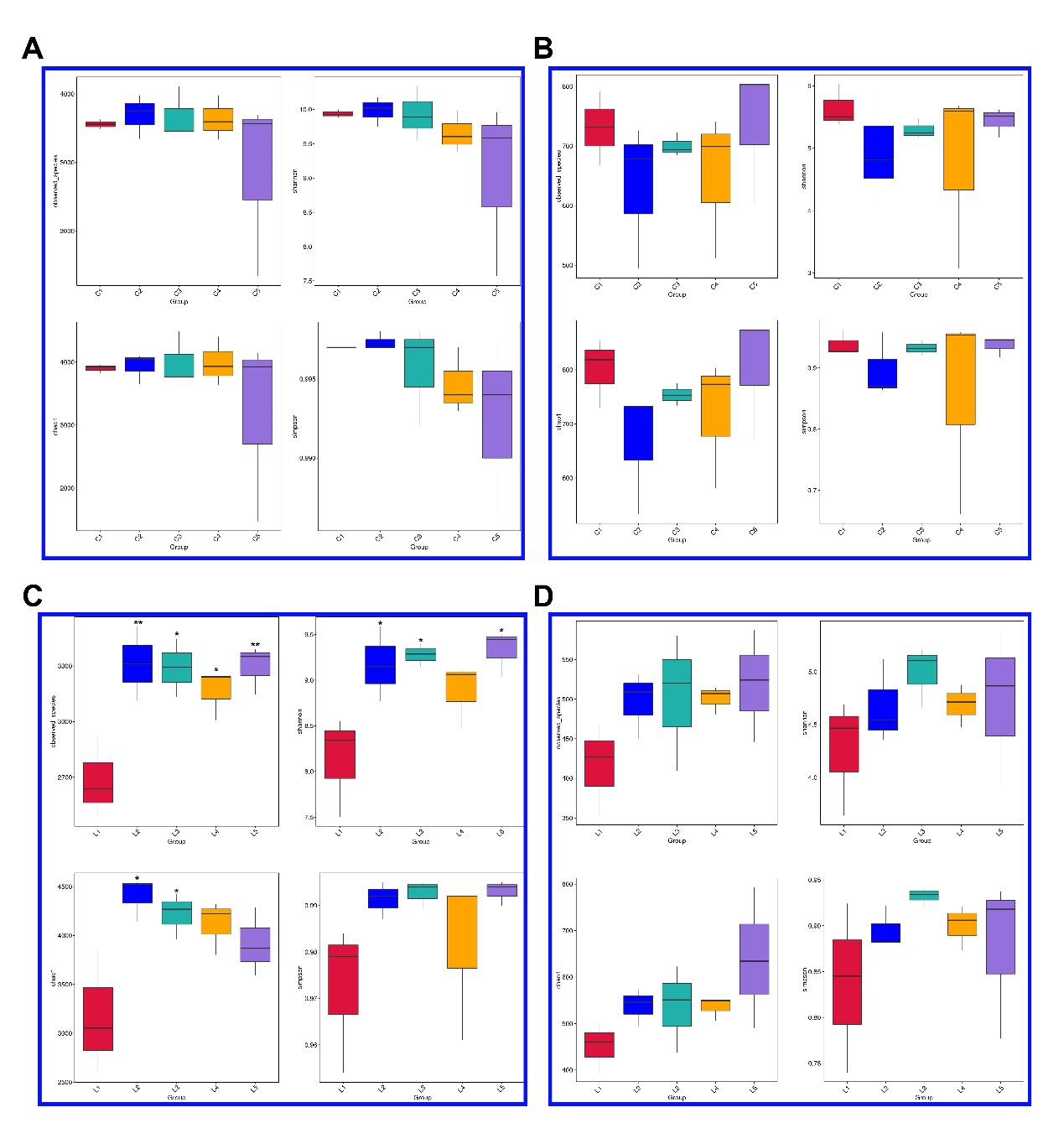


Figure S1. Response of microbial alpha-diversity to the inoculated strains. A-B: The corresponding parameters related to the bacterial (A) and fungal (B) community alpha-diversity of salinized soil. C-D: The corresponding parameters related to the bacterial (C) and fungal (D) community alpha-diversity of acid soil.


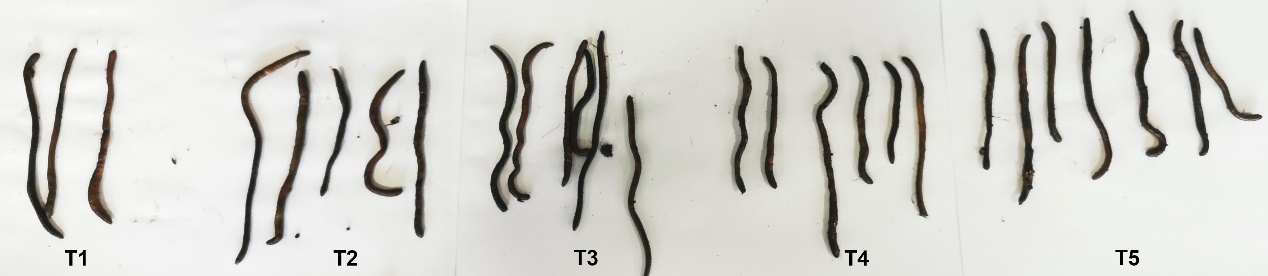


Figure S2. The number of earthworm in the salinized soil.


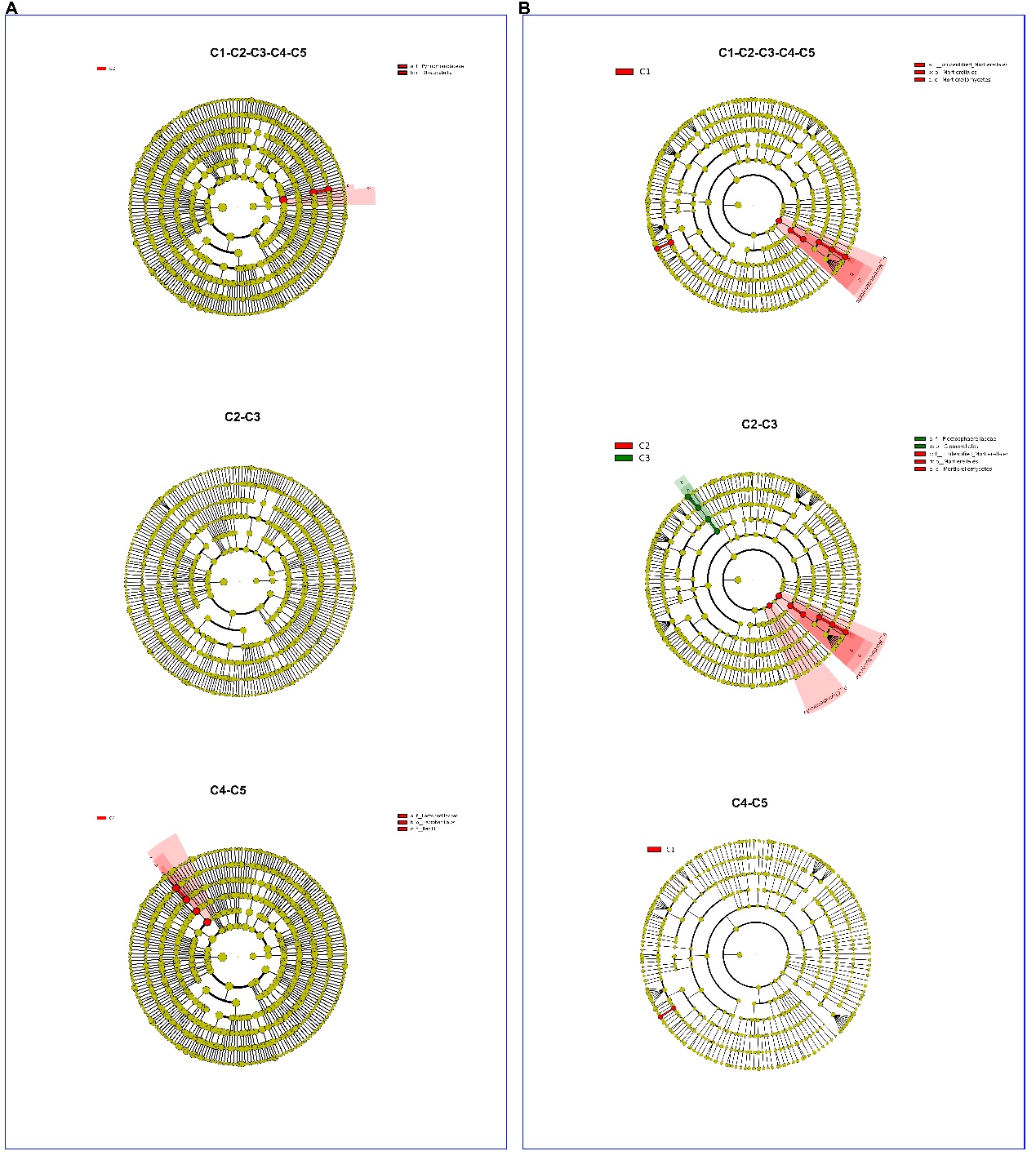


Figure S3. Lefse analysis to identify microbial biomarkers in each treatment of salinized soil. A-B: The results of bacterial (A) and fungal (B) communities. The yellow node represents that the species is not significantly enriched, and the nodes of other colors represent that the species are enriched in the corresponding treatment.


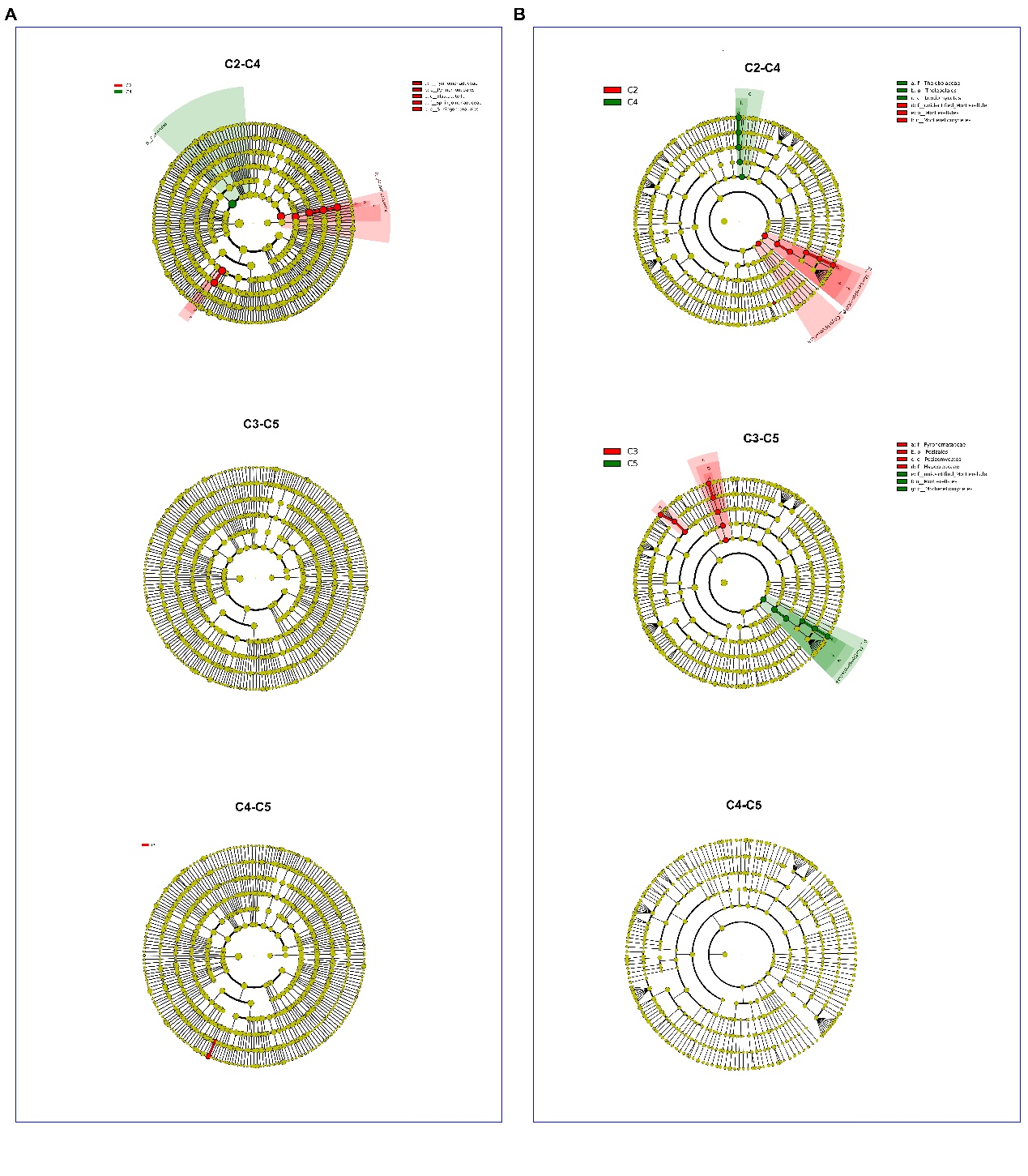


Figure S4. Lefse analysis to identify microbial biomarkers between different combinations of inoculated strains in salinized soil. A-B: The results of bacterial (A) and fungal (B) communities. The yellow node represents that the species is not significantly enriched, and the nodes of other colors represent that the species are enriched in the corresponding treatment.


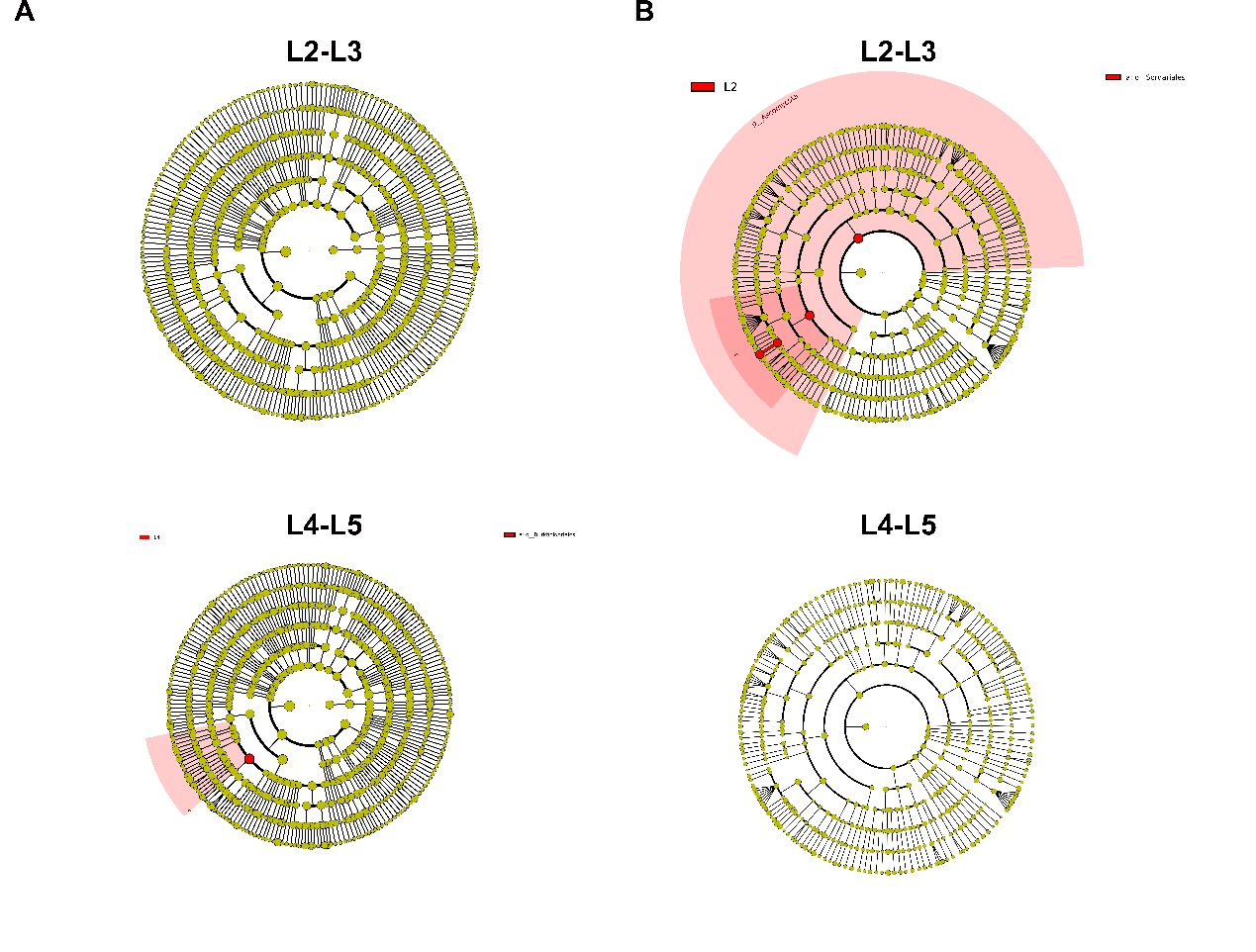


Figure S5. Lefse analysis to identify microbial biomarkers between different combinations of inoculated strains of acid soil. A-B: The results of bacterial (A) and fungal (B) communities. The yellow node represents that the species is not significantly enriched, and the nodes of other colors represent that the species are enriched in the corresponding treatment.


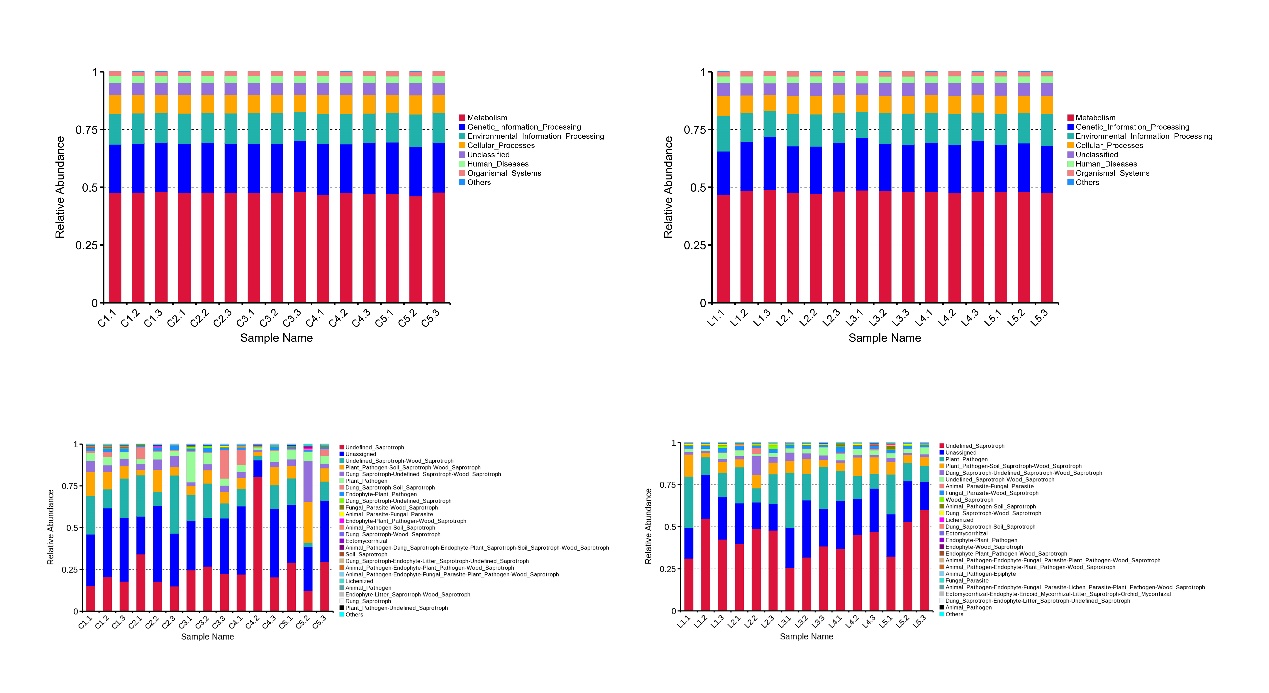


Figure S6. Distribution of bacterial and fungal function in the barrier soils. A-B: Bacterial function distribution in the salinized (A) and acid (B) soil. C-D: Fungal function distribution in the salinized (A) and acid (B) soil.


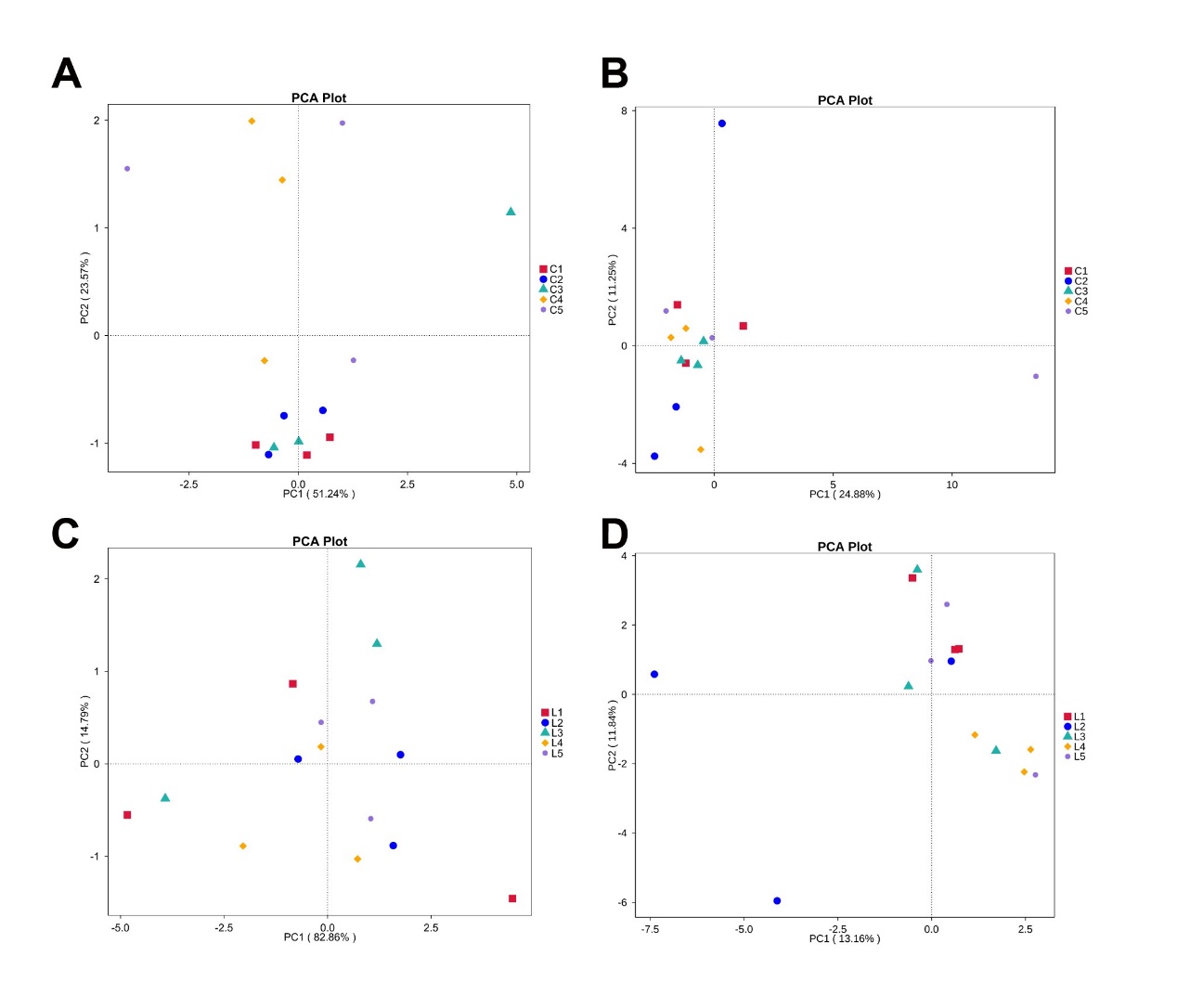


Figure S7. PCA to evaluate the function of bacterial or fungal community in the barrier soils. A-B: The function of bacterial (A) and fungal (B) communities in the salinized soil. C-D: The function of bacterial (C) and fungal (D) communities in the acid soil.
